# Gene Regulation in Primates Evolves under Tissue-Specific Selection Pressures

**DOI:** 10.1371/journal.pgen.1000271

**Published:** 2008-11-21

**Authors:** Ran Blekhman, Alicia Oshlack, Adrien E. Chabot, Gordon K. Smyth, Yoav Gilad

**Affiliations:** 1 Department of Human Genetics, University of Chicago, Chicago, Illinois, United States of America; 2 Walter and Eliza Hall Institute of Medical Research, Parkville, Victoria, Australia; University of Oxford, United Kingdom

## Abstract

Regulatory changes have long been hypothesized to play an important role in primate evolution. To identify adaptive regulatory changes in humans, we performed a genome-wide survey for genes in which regulation has likely evolved under natural selection. To do so, we used a multi-species microarray to measure gene expression levels in livers, kidneys, and hearts from six humans, chimpanzees, and rhesus macaques. This comparative gene expression data allowed us to identify a large number of genes, as well as specific pathways, whose inter-species expression profiles are consistent with the action of stabilizing or directional selection on gene regulation. Among the latter set, we found an enrichment of genes involved in metabolic pathways, consistent with the hypothesis that shifts in diet underlie many regulatory adaptations in humans. In addition, we found evidence for tissue-specific selection pressures, as well as lower rates of protein evolution for genes in which regulation evolves under natural selection. These observations are consistent with the notion that adaptive circumscribed changes in gene regulation have fewer deleterious pleiotropic effects compared with changes at the protein sequence level.

## Author Summary

It has long been hypothesized that in addition to structural changes to proteins, changes in gene regulation might underlie many of the anatomic and behavioral differences between humans and other primates. However, to date, there are only a handful of examples of regulatory adaptations in humans. In this work, we present a genome-wide study of gene expression levels in livers, kidneys, and hearts from three species: humans, chimpanzees, and rhesus macaques. These data allowed us to identify genes and entire pathways in which regulation evolved under natural selection and therefore are likely to be functionally important. Our results provide some of the first examples of pathways that have been remodeled specifically in the human lineage. In particular, we find that the regulation of a large number of genes involved in metabolic pathways evolved under lineage-specific directional selection. This result is intriguing, because, in addition to the obvious cognitive and linguistic differences between humans and non-human apes, a clear lifestyle shift between us and other primates can be found in our diet. We also found evidence for tissue-specific selection pressures on gene regulation, an observation that provides strong support to the notion that adaptive circumscribed changes in gene regulation have fewer deleterious pleiotropic effects compared with changes at the protein sequence level.

## Introduction

A central goal of evolutionary biology is to elucidate the genetic architecture of adaptation. In humans, in particular, this question is of interest both for what it will reveal about human specific traits [1]–[4] and because of the emerging links between adaptation and disease susceptibility [5],[6].

A long standing hypothesis is that changes in regulation play an important role in adaptive evolution, notably in primates [7]–[11]. Consistent with this theory, the past decade of research has yielded an increasing number of cases where regulatory changes contribute to species-specific adaptations and to reproductive isolation [8], . Nonetheless, to date, there are still only a handful of examples of regulatory adaptations in primates. A better understanding of the evolutionary forces influencing gene regulation in primates is not only of interest in an evolutionary context but also promises to shed light on the contribution of regulatory variation to human diseases [18]. Indeed, while the main focus of disease susceptibility studies has been on coding regions [19], a number of recent association studies of complex human diseases identified candidate loci in regulatory regions, or in intergenic regions, which are thought to have a function in gene regulation (e.g., references [20]–[23]). More generally, mutations in putative regulatory regions have been associated with well over 100 human phenotypes including diverse aspects of behavior, physiology and disease (reviewed in references [24] and [25]).

One approach to study the evolution of gene regulation is by studying variation in gene expression levels within and between populations or species. The challenge is then to use comparisons of variation within and between populations to distinguish between neutral changes in gene expression and patterns that are consistent with natural selection [26]. Ideally, one would want to partition the observed variation in gene expression into its genetic and non-genetic (e.g., environmental and genetic by environment interaction) components in order to study the genetic basis for variation in gene expression without the confounding effects of environmental variation. In model organisms, minimizing the difference in environment between samples helps to reduce the environmental variance, and mutation accumulation studies provide estimates of the neutral mutational variance in gene expression [27]–[29]. However, studying phenotypic evolution in primates is more difficult in this respect, because key experiments often cannot be performed to distinguish between competing hypotheses or to estimate important parameters (such as the neutral mutational variance). Moreover, material is often scarce, leading to largely unknown and uncontrolled environmental variance between samples. These limitations are particularly problematic for dynamic, environmentally sensitive traits like gene expression. In addition, until recently, most inter-primate studies of gene expression used microarrays that were designed based on the genomic sequences of only one species (“single-species arrays”), and as a result, their inter-species expression estimates were confounded by the effect of sequence mismatches on hybridization intensity [26],[30],[31].

Perhaps due to the difficulties discussed above, the first few studies that have examined the selection pressures that shape gene expression profiles in humans and close evolutionary relatives resulted in somewhat conflicting conclusions [32]–[38]. To address this, we previously developed a pilot multi-primate cDNA microarray, containing probes for 1056 genes expressed in human liver, which allows one to accurately estimate expression differences between species [31]. Using this pilot array, we estimated gene expression differences between liver samples from humans, chimpanzees, orangutans and rhesus macaques, and found that, consistent with observations in model organisms [27]–[29],[39], there was little evidence for change in expression levels across species for most genes, suggesting widespread stabilizing selection. Nonetheless, the regulation of a subset of genes appeared to have evolved under positive (directional) selection in the human or chimpanzee lineages [11].

Here, we used a second generation of the multi-species array, with probes for 18,109 orthologous genes from human, chimpanzee, and rhesus macaque, to estimate variation in gene expression within and between species in livers, kidneys, and hearts. Using this comparative expression data, we identified genes whose regulation likely evolves under natural selection, including a large number of transcription factors. We also identified specific regulatory pathways, notably metabolic pathways, which have likely been remodeled exclusively in the human lineage.

## Results

In order to facilitate a comparison of gene expression between three primate species, we designed a novel genome-wide multi-species NimbleGen microarray. This microarray contains orthologous probes from human, chimpanzee, and rhesus macaque, thus allowing a comparison of gene expression levels within and between these primate species without the confounding effect of sequence mismatches on hybridization intensities [31]. The microarray contains probes from 18,109 genes (Table S1), with the vast majority of genes represented by seven probes per gene per species, for a total of ∼370,000 probes (see Methods).

We used the multi-primate microarray to measure gene expression levels in livers, kidneys, and hearts from six individuals from each of the three species (Table S2), in two technical replicates, for a total of 108 microarray hybridizations (see Figure S1 for an illustration of the study design). We performed extensive quality control analyses to ensure that the data quality is high (see Text S1 and Figures S1, S2, S3, S4, S5, S6, S7, S8, S9, S10, and S11 for details). Our comparative gene expression data allows us to study variation in gene expression within and between tissues and species.

### Gene Expression Differences between Tissues and Species

We used a linear mixed-effects model to analyze the background-corrected, normalized probe-level data for each tissue. Our gene-wise model was designed with fixed effects for species, sequence mismatches, and probes, and a random effect for individuals (see Methods). As a first step of our analysis, we used estimates from the linear model to identify genes that are differentially expressed between tissues (z-statistic, FDR<0.01). We observed a consistent pattern whereby, in all species, the number of differentially expressed genes is lowest in the comparison between kidney and heart (Table 1).

**Table 1:** Numbers of differentially expressed (DE) genes between tissues and species (from a total of 17,231 orthologous genes in human, chimpanzee, and rhesus macaque, which passed quality controls and were included in the analysis).

DE between tissues (within species)	liver-kidney	liver-heart	kidney-heart
Human	2810	2662	2124
Chimpanzee	2590	2894	2222
Rhesus Macaque	2522	2768	2215
**DE between species**	**Liver**	**Kidney**	**Heart**
Human vs. Chimpanzee	2809	3368	3197
Human vs. Rhesus Macaque	5525	6250	5545
Chimpanzee vs. Rhesus Macaque	4871	6270	5021

Next, we used likelihood ratio tests within the framework of the linear model to identify differentially expressed genes between pairs of species, choosing a cutoff of 10 for the χ^2^-distributed likelihood ratio test statistic (which corresponds to global FDR<0.006 across all comparisons in Table 1). As expected, in all tissues, the number of differentially expressed genes is (roughly two-fold) smaller between human and chimpanzee than between human (or chimpanzee) and rhesus macaque. Interestingly, in liver and heart, we find more differentially expressed genes between human and rhesus macaque than between chimpanzee and rhesus macaque (whereas in kidney the numbers are comparable). Also, while the number of differentially expressed genes between species is smaller in liver compared with the other two tissues (regardless of the species), the magnitude of expression change is slightly larger in the liver: for example, while 15% (421) of the genes differentially expressed between the human and chimpanzee livers are different by more than 1.5-fold, this is the case for only 9% (303) and 13% (415) of the differentially expressed genes in kidney and heart, respectively. (We observed similar patterns for genes differentially expressed between the other pairs of species.) Thus, a first overview of the inter-species gene expression pattern across the three tissues suggests a marginally higher rate of regulatory evolution in the liver, notably in humans. This observation is consistent with previous results [40].

**Figure 1:**
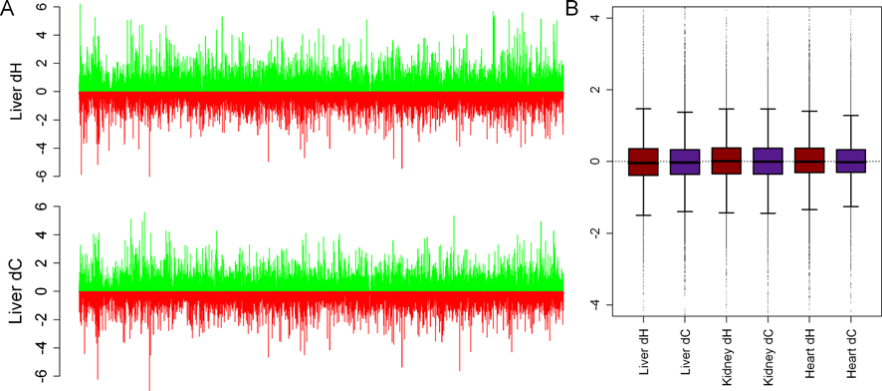
Estimates of lineage-specific expression changes. A. Increase (green bars) and decrease (red bars) of gene expression levels in the human (d_H_, top) and chimpanzee (d_C_, bottom) lineages are plotted. B. Box plots of the estimated expression changes (y-axis) along the human (red) and chimpanzee (purple) lineages in liver, kidney, and heart (x-axis).

In order to infer lineage-specific expression changes, we used the expression level in rhesus macaque as an estimate of the gene expression level in the common ancestor of human and chimpanzee. Based on this estimate, we calculated lineage-specific changes in gene expression in the human and chimpanzee lineages (see Text S1). Contrary to previous reports, we do not find evidence for ‘accelerated’ gene expression evolution in either lineage. Indeed, the magnitude of lineage-specific change is higher in human compared to chimpanzee in 47.9%, 50.7% and 51.7% of genes in liver, kidney, and heart, respectively (Figure 1A). Similarly, we find no evidence for bias towards elevated expression levels in either lineage: the proportion of genes with elevated expression level compared to the estimate of the ancestral gene expression level is 0.46 and 0.47, for human and chimpanzee, respectively, in liver, 0.51 and 0.50 in kidney, and 0.49 and 0.48 in heart (Figure 1B). As our estimate of the ancestral expression level relies on the unrealistic assumption that there has been no change of expression level in rhesus macaque or in the common ancestor of human and chimpanzee, we confirmed that similar patterns of lineage-specific expression changes are seen when we retain only genes for which the rhesus macaque expression level is an intermediate between the human and chimpanzee expression levels (i.e., when deviations from this assumption will have a smaller effect; see Figures S9 and S10).

### Gene Expression Differences in Chromosomal Rearrangements

We examined whether inter-species differentially expressed genes are more likely to be located in proximity to known chromosomal rearrangements, as has been observed previously in a smaller dataset [35]. The largest chromosomal rearrangement that occurred in the human lineage is the fusion of two independent great ape chromosomes that created the human chromosome 2 [41]. On chromosome 2, we find a slight enrichment of genes that are differentially expressed between human and chimpanzee in heart (by FET; one tailed *P* = 0.02; assuming that differentially expressed genes are randomly distributed in the genome). Moreover, using the estimated position of the fusion point [42] and by considering only genes located on chromosome 2, we find that genes that are differentially expressed between human and chimpanzee heart samples are enriched within a region of 10 Mb on either side of the fusion point (one tailed *P* = 0.03).

To study this further, we focused on seven other known large-scale chromosomal rearrangements between humans and chimpanzees [43] (Table S6), and tested whether genes that are differentially expressed between the species are enriched in the area flanking the breakpoints (within 10 Mb). We found similar patterns in two of the seven rearrangements (on chromosome 16: genes differentially expressed between the species in liver, *P* = 0.002; and on chromosome 18: genes differentially expressed between the species in heart, *P* = 0.04). Although the patterns are weak, taken together, these results are consistent with previous observations [35] and suggest a role for large-scale chromosomal rearrangements in the evolution of gene regulation.

### Constraint on Gene Expression Levels

Our next analysis aimed at finding genes whose expression profiles are consistent with the action of natural selection on gene regulation. As discussed in the introduction, we are unable to explicitly test a null model of no selection due to uncertainty about salient parameters in primates. Instead, we identified genes whose regulation has likely evolved under evolutionary constraint by using a heuristic approach based on the expectation that gene expression levels under constraint will vary little within and between species.

As a first step, we ranked genes by their estimated between-individual variance for each tissue. Based on the ranked distribution of the estimated variance across genes, we classified genes as having high or low between-individual variance (Figures S12 and S13, see Methods). We excluded 17–26% of genes (depending on the tissue) with very low absolute intensity values, as these genes may have low expression variance between individuals simply because they are not expressed, or because their probes do not hybridize effectively (see Methods and Figure S14). Of the remaining genes, low between-individual variance in gene expression (i.e., low within-species variance) may reflect constraint on gene regulation.

Indeed, among genes with low between-individual variance - regardless of the tissue - we find enrichments (unadjusted FET *P*<0.05; see Text S1) of genes that are traditionally defined as ‘housekeeping’, genes involved in metabolic pathways, and transcription factors. Among genes with high between-individual variance, we find enrichments of genes associated with different human diseases (Table S3).

**Figure 2:**
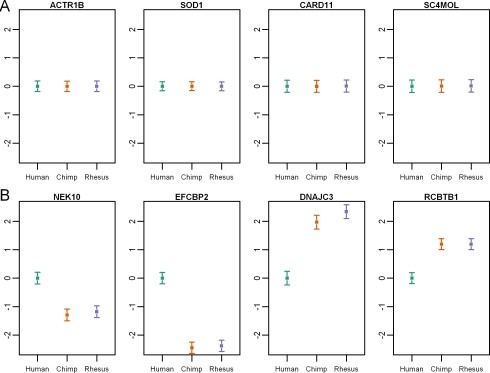
Examples of expression patterns that are consistent with the action of natural selection. Liver expression profiles from the three species are plotted for genes whose regulation has likely evolved under stabilizing (A) or directional (B) selection. In all panels, the mean (±s.e.m) log expression level (y-axis) of each species (x-axis) is plotted relative to the human value.

The next step was to identify genes whose expression patterns between as well as within species are consistent with evolutionary constraint on gene regulation. To do so, we used an approach similar to the one used by Gilad et al., (2006) [11], namely, we ranked genes by the summary of the evidence for stabilizing selection within and between species. Our approach relies on the expectation that genes whose expression levels remained constant within and between species will be enriched with genes whose regulation evolves under stabilizing selection (see Methods for more details as well as Figure 2A for examples of such patterns).

**Figure 3:**
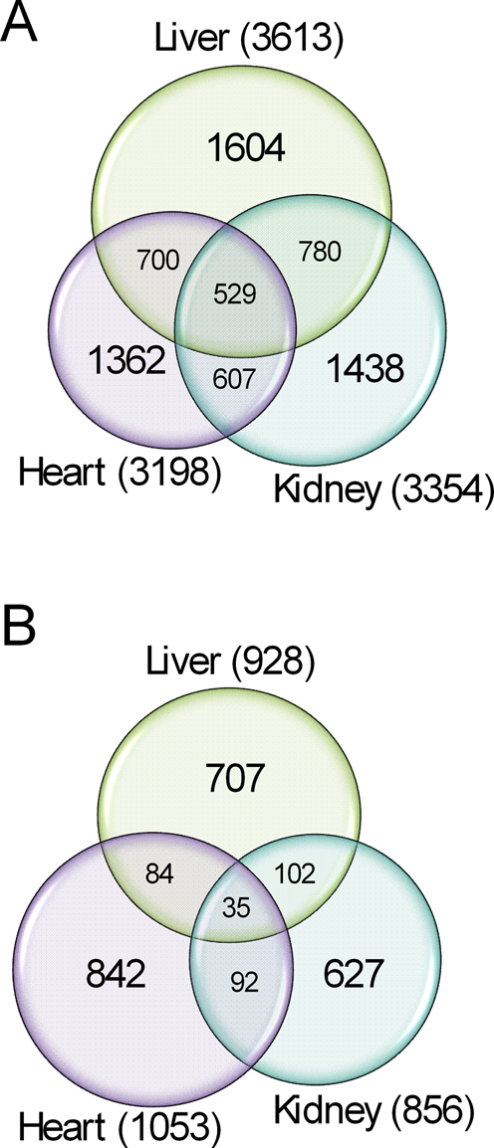
Comparison of data across tissues. Venn diagrams showing the number of genes whose regulation likely evolved under stabilizing (A) and directional (B) selection in liver, kidney, and heart.

Using this approach, we identified 3613, 3354, and 3198 genes with constrained expression patterns within and between species in liver, kidney, and heart, respectively (Figure 3; Table S1). The overlap of such genes across all three tissues is highly significant (529 genes, compared with an expected overlap of 118 genes if results across the three tissues were independent), consistent with our intuition that a large number of genes have important functions in multiple tissues.

As expected, among genes with constrained expression patterns within and between species, we find enrichments of ‘housekeeping’ genes, metabolic genes, and transcription factors, regardless of the tissue (Table 2 and Table S7). We also find enrichments for genes in which somatic or germline mutations have been causally implicated in cancer (Table 2). When we looked for specific pathways that might be enriched for genes whose regulation is constrained (see our discussion regarding multiple testing in Text S1), we found a number of pathways that are associated with complex human diseases in all tissues (Table 2) as well as the adherens junction pathway, methionine metabolism and genes involved in cell cycle in liver; reductive carboxylate cycle (CO_2_ fixation) and ribosomal genes in kidney; and TGF-beta signaling pathway and proteasome genes in heart.

**Table 2:** Functional categories (top, *italics*) and pathways (bottom) that are enriched among genes whose regulation evolves under stabilizing selection.

Tissue	Category	*P*-value
*Liver (functional categories)*	*Housekeeping*	*<10^−13^*
	*Metabolic (GO)*	*<10^−9^*
	*Transcription factors (GO)*	*<10^−4^*
	*Transcription factors (validated)*	*<10^−4^*
	*Associated with cancer*	*<10^−3^*
Liver (pathways)	Methionine metabolism	<10^−3^
	Complement and coagulation cascades	<10^−3^
	Adherens junction	<10^−3^
	Cell cycle	0.003
	TGF-beta signaling pathway	0.007
*Kidney (functional categories)*	*Housekeeping*	*<10^−7^*
	*Transcription factors (GO)*	*0.002*
	*Transcription factors (validated)*	*0.013*
	*Metabolic (GO)*	*0.043*
Kidney (pathways)	Amyotrophic lateral sclerosis (ALS)	<10^−3^
	Reductive carboxylate cycle (CO2 fixation)	0.020
	Ribosome	0.025
	Neurodegenerative Diseases	0.029
	Pathogenic Escherichia coli infection - EHEC	0.033
*Heart (functional categories)*	*Metabolic (GO)*	*<10^−9^*
	*Transcription factors (validated)*	*<10^−7^*
	*Housekeeping*	*<10^−6^*
	*Transcription factors (GO)*	*<10^−5^*
	*Associated with cancer*	*<10^−3^*
Heart (pathways)	Proteasome	<10^−3^
	Focal adhesion	<10^−3^
	Chronic myeloid leukemia	0.001
	Pancreatic cancer	0.002
	TGF-beta signaling pathway	0.003
*LKH vs. L|K|H (functional categories)*	*Housekeeping*	*<10^−4^*
	*Transcription factors (validated)*	*0.034*
	*Transcription factors (GO)*	*0.043*
LKH vs. L|K|H (pathways)	Chronic myeloid leukemia	0.004
	Thyroid cancer	0.005
	Pancreatic cancer	0.008
	TGF-beta signaling pathway	0.008
	Proteasome	0.009

*P*-values were calculated using a Fisher exact test. Note that only specific GO categories were tested in this analysis (see Methods for more details).

### Directional Selection on Gene Regulation

Using a similar approach, we also looked for expression patterns that are consistent with directional selection on gene regulation, namely, a significant lineage-specific shift in gene expression level combined with low within-species variance [31]. For example, we expect an enrichment of genes whose regulation evolves under directional selection in humans among the group of genes whose expression levels are constant within and between the non-human primates, but whose expression levels were significantly elevated or reduced exclusively in the human lineage (see Figure 2B and Figure S15 for examples of such patterns).

Using this approach, we found 928, 856, and 1053 genes with constant expression levels in the non-human primates and a significantly different expression level exclusively in humans, in liver, kidney, and heart, respectively (Figure 3; Table S1). The overlap of such genes across tissues is relatively small, an observation that may reflect the flexibility of adaptation through changes in gene regulation (see Discussion).

In agreement with our previous observations for only 907 genes [11], we find an enrichment of transcription factors among genes whose regulation likely evolved under directional selection in humans, regardless of the tissue (Table 3 and Table S7). We find similar enrichments for genes that belong to the focal adhesion, adherens junction, and tight junction pathways. In addition, we find tissue-specific enrichments of genes associated with different metabolic pathways in the human liver; glycerolipid metabolism, inositol phosphate metabolism, and riboflavin metabolism in human kidney; and fatty acid metabolism as well as genes associated with metabolic syndromes and dyslipidemia in the human heart (Table 3 and Table S7).

**Table 3:** Functional categories (top, *italics*) and pathways (bottom) that are enriched among genes whose regulation evolves under directional selection.

Tissue	Category	*P*-value
**Liver, human higher**	*Dyslipidemia*	*0.004*
	*Transcription factors (GO)*	*0.045*
	Focal adhesion	0.001
	ECM-receptor interaction	0.008
	Tight junction	0.011
	PPAR signaling pathway	0.028
	Glutamate metabolism	0.033
**Liver, human lower**	*Metabolic (GO)*	*<10^−3^*
	*Neuroactive ligand-receptor interaction*	*0.005*
	Adherens junction	0.015
	SNARE interactions in vesicular transport	0.026
	Neurodegenerative Diseases	0.034
**Kidney, human higher**	*No enrichments found*	*N/A*
	Glycerolipid metabolism	0.019
**Kidney, human lower**	*Transcription factors (validated)*	*0.045*
	Inositol phosphate metabolism	0.032
	Adherens junction	0.039
	Riboflavin metabolism	0.040
**Heart, human higher**	*Associated with metabolic disorders*	*0.044*
	*Dyslipidemia*	*0.048*
	*Transcription factors (GO)*	*0.049*
	Leukocyte transendothelial migration	0.006
	Chronic myeloid leukemia	0.007
	Tight junction	0.007
	Thyroid cancer	0.011
	Glycan structures - biosynthesis 1	0.017
**Heart, human lower**	*No enrichments found*	*N/A*
	Citrate cycle (TCA cycle)	<10^−4^
	Oxidative phosphorylation	<10^−3^
	Valine, leucine and isoleucine degradation	<10^−3^
	Reductive carboxylate cycle (CO2 fixation)	0.001
	Fatty acid metabolism	0.003
**Directional selection in any tissue (human higher or lower)**	*Transcription factors (GO)*	*0.007*
	*Metabolic (GO)*	*0.012*
	Tight junction	0.001
	Citrate cycle (TCA cycle)	0.002
	Adherens junction	0.014
	Glutamate metabolism	0.015
	Thyroid cancer	0.034

*P*-values were calculated using a Fisher exact test. Note that only specific GO categories were tested in this analysis (see Methods for more details).

**Figure 4:**
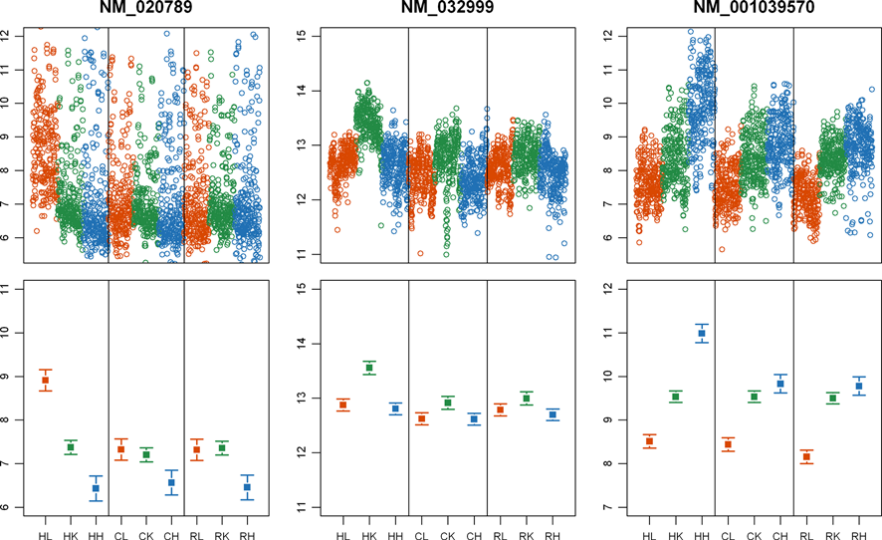
Directional selection on gene regulation in humans affects metabolic pathways. The interaction network was generated using the Ingenuity Pathway Analysis (IPA) tool (version 6.0). All shaded nodes represent genes whose regulation evolves under directional selection. Transcription factors are shaded in orange. Specific metabolic functions that are associated with the individual genes are listed.

In order to gain further insight into the phenotypes that might be affected by directional selection on gene regulation in humans, we used the Ingenuity pathway analysis tool (http://www.ingenuity.com/) to explore known interactions between genes. Figure 4 illustrates the top interaction network generated using genes whose regulation is under directional selection in liver. As can be seen, this network is enriched with transcription factors and genes with metabolic functions. The phenotypes that may be affected by the regulatory perturbation of this network include carbohydrate metabolism, lipid metabolism, and calcium signaling. Indeed, selection on metabolic related pathways, and in particular on calcium signaling, is particularly intriguing given the marked shift in diet that occurred during human evolution.

When we performed a similar analysis to identify genes with constant expression levels in rhesus macaques and humans and a significantly different expression level exclusively in chimpanzees, we found 686, 774, and 761 such genes in liver, kidney, and heart, respectively (Figure S16). Thus, our observations suggest that, regardless of the tissue, fewer genes underwent directional selection at the regulatory level in chimpanzee compared to human (74%, 90%, and 72% in liver, kidney, and heart, respectively). Furthermore, in contrast to our observations in humans, we did not find an enrichment of transcription factors among genes whose regulation has likely evolved under directional selection in the chimpanzee (Table S4; in chimpanzee liver, we found a slight under-representation of transcription factors among such genes; by FET, *P* = 0.04).

### Tissue-Specific Selection Pressures

**Figure 5:**
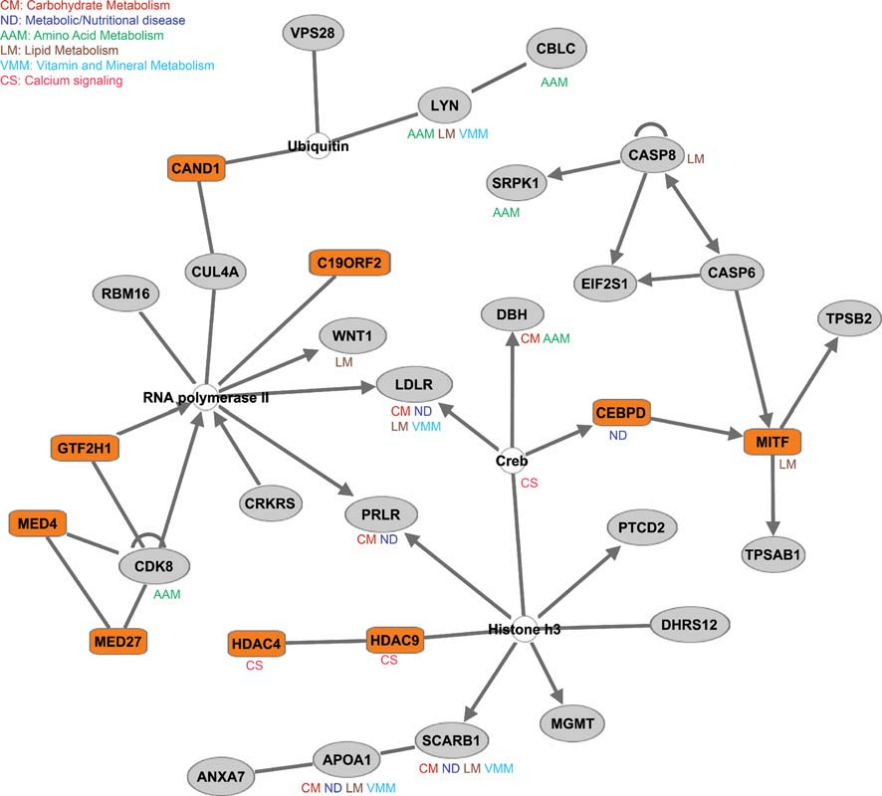
Tissue-specific selection on gene regulation. Examples of expression patterns that are consistent with the action of directional selection on gene regulation in the human liver (A), kidney (B), or heart (C) and the action stabilizing selection on gene regulation in the other two tissues. In the top panels, we plot the normalized log-expression intensities of all the probes for these genes from all relevant hybridizations and in the bottom panel the estimated relative log expression levels (±s.e.m). On the x-axis, HL stands for expression results from human liver; HK - human kidney; HH - human heart; CL - chimpanzee liver; CK - chimpanzee kidney; CH - chimpanzee heart; RL - rhesus macaque liver; RK - rhesus macaque kidney; RH - rhesus macaque heart.

The comparison of gene expression patterns within and between tissues and species also allowed us to examine tissue-specific selection pressures on gene regulation. In principle, one might argue that there is reasonable evidence for tissue-specific selection pressure in every case where a pattern that is consistent with the action of natural selection is inferred based on the expression data from one tissue but not others. However, since our inference is based on ranking genes by a summary of their expression level variation within and between species, lack of evidence for natural selection using our approach cannot be taken as good evidence for no selection. We therefore applied more stringent criteria, using the approaches described above to identify genes for which we have evidence for distinct types of selection on gene regulation in different tissues. Examples of such patterns are given in Figure 5 for genes whose regulation appears to evolve under directional selection in one human tissue yet whose regulation seems to be under stabilizing selection in the two other tissues. By combining such information across tissues and species, we found 48, 65, and 74 genes whose regulation evolves under stabilizing selection in two tissues, and under directional selection in the human liver, kidney, and heart, respectively (Table S1). Similarly, we found 35, 45, and 43 genes whose regulation evolves under stabilizing selection in two tissues, and under directional selection in the chimpanzee liver, kidney, and heart, respectively. Thus, even though we imposed highly stringent criteria, we found a clear signature of tissue-specific natural selection on gene regulation for an appreciable number of genes.

### Selection on Protein Coding Regions

**Figure 6:**
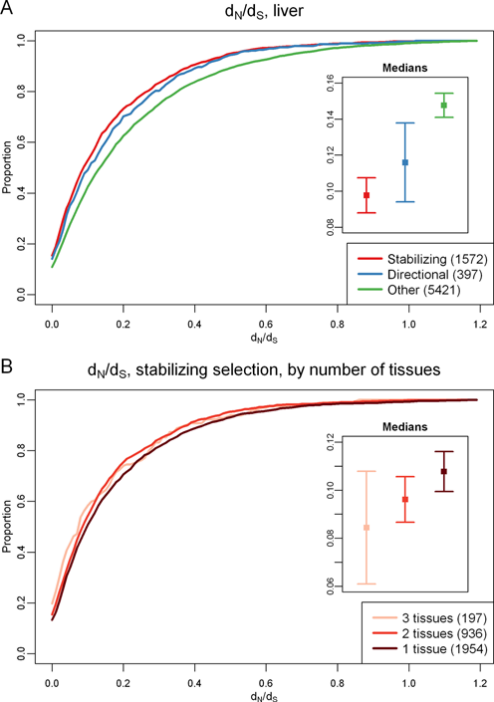
Protein evolution and selection on gene regulation. Cumulative distributions of d_N_/d_S_ values (x-axis) of (A) genes whose regulation evolved under stabilizing selection in the liver (red), directional selection in the liver (blue), or for which we do not have evidence for selection on gene regulation in the liver (green), and (B) genes whose regulation evolved under stabilizing selection in one (pink), two (red), or three (black) tissues. The smaller panels show the d_N_/d_S_ medians in the three groups. The error bars are 95% confidence intervals calculated using bootstrapping (1000 repetitions).

Finally, we examined the relationship between selection on gene regulation and selection at the protein coding level. To address this question, we used d_N_/d_S_ ratios as a measure of protein evolution, i.e., the ratio of the rates of non-synonymous to synonymous substitutions (see Text S1 for more details). Regardless of the tissue, we observed significantly lower d_N_/d_S_ values for genes whose regulation evolves under natural selection (either stabilizing or directional) compared with genes for which we did not find evidence for selection at the gene expression level (by permutation, all *P*<0.022; see Figures 6A and S17 for liver gene expression data and Table S5 for all tissue-specific comparisons). Moreover, we observed significantly lower d_N_/d_S_ values for genes whose regulation evolves under stabilizing selection in all three tissues compared with genes for which we have evidence for stabilizing selection on gene expression levels only in one tissue (by permutation; *P* = 0.024; see Figure 6B).

## Discussion

We used a novel genome-wide multi-species microarray to study variation in gene expression levels within and between tissue samples from humans, chimpanzees, and rhesus macaques. Using these data, we identified gene expression patterns within and between species, which are consistent with the action of natural selection on gene regulation. Previous studies have done so by testing for deviations from a specified null model [38],[44],[45]. However, such an approach relies on a number of parameter estimates about which there is considerable uncertainty in primates. Instead of specifying an explicit model, we took what is often termed an ‘empirical approach’ [46],[47], namely, we used statistical analyses to rank genes based on their pattern of evolutionary change among the three species and focused on those at the top of the list as the most promising candidates.

Such empirical approaches are widely used in the analysis of sequence data to scan for recent targets of natural selection, for example by ranking genomic regions by their F_st_ values between populations, by the extent of haplotype sharing, or by the magnitude of deviations from the site frequency spectrum expected under the standard neutral model (e.g., [46],[47]). In all cases, the rationale is that genomic regions at the top of the list are expected to be enriched with targets of recent natural selection. It is recognized, however, that not all genomic regions at the top of list (regardless of the cutoff chosen) are indeed targets of natural selection, and conversely, not all true targets of natural selection will be at the top of the list [48].

In our case, we relied on the expectation that genes whose regulation evolves under stabilizing selection should have very little variation in gene expression levels within as well as between species. Similarly, genes whose regulation has evolved under directional selection in humans are expected to have a different expression level in humans compared with the other species, while maintaining low variance between human individuals (while a shift in the mean gene expression level coupled with increased between individual variance is also consistent with a lineage-specific relaxation of evolutionary constraint).

The cutoffs that we chose for the classification of genes whose regulation evolved under different selection pressures are objective (based on the ranked distribution of between-individual variance; see Text S1), but arbitrary. Indeed, while it is clear that there is better evidence for selection on gene regulation for genes at the top of the lists, it is difficult to choose a cutoff below which the evidence for selection is no longer compelling. Thus, although throughout this paper we refer to genes whose regulation has likely evolved under natural selection, it is important to remember that the basis for our inference is the ranking of expression level variation within and between species, not direct evidence for the presence or absence of natural selection. Moreover, low expression divergence may result from low mutational input rather than the action of natural selection. As we cannot directly study the mutational input for gene expression variation in primates, we are unable to offer specific insight into which levels of gene expression divergence indicate the action of natural selection rather than low mutational input.

Notably, however, we confirmed that our qualitative conclusions are robust with respect to the specific cutoffs chosen, including the enrichment of transcription factors and metabolic genes among genes whose regulation is inferred to evolve under selection, as well as the correlation between selection on gene regulation and evolutionary constraint at the protein coding level.

### Genetic or Environmental Differences?

An important caveat of studies of primate tissues, including the current study, is that we cannot stage the primate tissues that we work with, or control for environmental effects on gene expression. In addition, due to the difficulty of obtaining tissue samples from chimpanzees, we could not perfectly balance the study design with respect to sex (see Table S2), and yet our sex-specific sample sizes are too small to explicitly take into account the effects of sex and sex-by-species interaction on gene expression levels. Similarly, while all our samples were obtained from adult individuals, we could not match the ages across species. Thus, although it is well known that gene expression levels are affected by age, sex, and different environments, in our analysis, we could not account for these effects.

We note, however, that variation in age, environment, and sex should generally result in an increase in gene expression variance between individuals. In our different analyses, we focused on genes with low between individual gene expression variance. In other words, we focused on genes that have highly constrained expression levels between individuals, even though the individuals were not controlled for age, sex, and environment. Our findings are therefore unlikely to be affected by the effects of environmental variation between individuals – although we may miss additional genes whose expression levels were perturbed by non-genetic effects.

In contrast, our findings may be affected by environmental variation between species, as it is likely that individuals from the same species share a more common environment than individuals from different species. For example, all non-human primate individuals may share certain aspects of their diet, which may be lacking from the diet shared by humans. Such species-specific environmental effects may contribute to the observed inter-species differences in gene expression and, in our study, would be indistinguishable from genetic effects.

### Mechanisms of Regulatory Change

Changes in regulatory elements may be more likely to underlie adaptive phenotypes if mutations in these elements produce circumscribed expression pattern changes. The rationale is that changes in gene regulation that are affecting limited number of cell types or tissues may result in fewer deleterious pleiotropic effects than might be expected when protein sequences are changed [49]–[51]. Several of our findings support this conjecture.

First, we observed a much smaller overlap across tissues of genes whose regulation evolved under directional compared with stabilizing selection. Second, we found evidence for tissue-specific selection pressures, whereby a gene's expression pattern may be consistent with directional selection in one tissue and stabilizing selection in the other tissues. Both of these observations are consistent with adaptive changes in regulatory elements that affect the expression patterns of individual genes in one tissue, without affecting gene functions and regulations in other tissues.

Third, we found evidence for a correlation between both stabilizing and directional selection on gene regulation and evolutionary constraint at the protein sequence level (note that this result is inconsistent with our previous observation, which was based on a much smaller number of genes [11]). This observation suggests that adaptation at the regulatory level occurs disproportionably in genes that are widely constrained at the protein sequence level. In other words, our results support the hypothesis that adaptation through changes in evolutionary constrained genes can occur by altering their regulatory patterns.

Moreover, we observed the lowest rates of protein evolution for genes whose regulation evolves under stabilizing selection in multiple tissues. These results support and refine previous observations of a correlation between gene expression breadth and rates of protein evolution [34],[52]. Indeed, while previous studies used gene expression as indication of function (i.e., when a gene is expressed in a given tissue it was concluded that it has a function in that tissue), here, we use tissue-specific stabilizing selection on gene regulation to indicate that a gene is functionally important in that tissue.

### Regulatory Evolution through Transcription Factors

A curious observation is that transcription factors appear to be enriched among genes whose expression profiles are consistent with the action of directional selection on gene regulation in humans, but not in chimpanzees. This result is consistent with our previous observation based on a much smaller number of genes, using a different array platform and using tissue samples from different human and chimpanzee individuals [11]. Evolution of gene regulation through transcription factors is an intuitively appealing mechanism, as a small change in a transcription factor expression level can affect the regulation of a large number of genes and result in a significant phenotypic effect [53].

While we cannot explain why this pattern is specific to humans, we note that the number of genes whose regulation evolves under directional selection is significantly larger in the human lineage compared with the chimpanzee lineage (in all tissues). This is in contrast to the pattern observed when we considered lineage-specific estimate of expression change for all genes (Figure 1), for which we find similar lineage-specific changes in gene expression for both human and chimpanzee. Thus, the difference in the overall number of genes whose regulation evolves under directional selection in humans and chimpanzees does not seem to have a technical explanation (i.e., it is unlikely an artifact). Instead, this difference between the patterns in human and chimpanzee may reflect a signature of regulatory propagation of the effects of directional selection on transcription factor regulation in humans.

### Regulatory Adaptations and Shifts in Diet

Our results provide some of the first examples of pathways that have likely been remodeled specifically in the human lineage. In particular, we find a signature consistent with the action of directional selection on gene regulation in genes involved in metabolic pathways in both humans and chimpanzees, with different pathways undergoing selection in each lineage. This result is intriguing because, in addition to the obvious cognitive and linguistic differences between humans and non-human apes, a clear life-style shift between us and other primates can be found in our diet. For example, we are the only primate to regularly consume cooked food, with the earliest unequivocal evidence for controlled use of fire dating to ∼400,000 years ago [54]. The digestion of cooked food, among other shifts in nutrition such as increased calcium intake and greater meat consumption, has led to a human diet that differs sharply from that of our close relatives [55]. Such changes are likely to have been accompanied by molecular adaptations [56]–[58], in particular, in relevant tissues such as liver and kidney.

### Summary

While we cannot directly study selection on gene regulation in primates, our comparative genomics expression data allowed us to identify a large number of genes and specific pathways with expression patterns within and between species that are consistent with the action of natural selection on gene regulation. Our observations raise interesting hypotheses regarding functional differences between humans and other primates, which may be subjected to further tests using cell line systems or model organisms. Finally, our results support the long standing hypothesis that changes in gene regulation have an important role in human evolution, and suggest that many adaptive regulatory changes in humans may be mediated through directional selection on transcription factor gene expression levels.

## Methods

### Multi-Species Array Design

All known human mRNA sequences were downloaded from the RefSeq database (www.ncbi.nih.gov/RefSeq) in August 2006 (RefSeq release 18). When multiple variants existed for the same gene, we considered only the longest available transcript. To find the non-human primate orthologous sequences for the human mRNAs, we downloaded the full genome sequences of chimpanzee (*Pan troglodytes*, March 2006 draft, panTro2) and rhesus macaque (*Macaca mulatta*, January 2006 draft, rheMac2) from the UCSC Genome Browser database (www.genome.ucsc.edu). We then used BLAT [59] to align the human mRNA sequences to the chimpanzee and rhesus macaque genomes. The BLAT algorithm allows one to align mRNA in blocks (corresponding to exons in this case), skipping the introns in the target genome. After filtering the matches by aligned sequence length (the numbers of “matching” aligned bases), we found chimpanzee and rhesus macaque orthologs for 18,109 human genes (complete 3-way alignments are available by request). We performed several quality controls to examine this alignment that are detailed in Text S1.

Based on our alignments, probes for the microarray were designed by NimbleGen (www.nimblegen.com). Within each gene, a set of up to 7 non-overlapping 60mer genic regions were chosen as probes from the human gene sequence (hereafter: a probe-set). The corresponding orthologous sequences in the other two genomes defined species-specific probes for chimpanzee and rhesus macaque. Hence, each probe-set consists of up to 7 species-specific probes that are aligned to different locations in the gene, and there are 3 species-specific versions for each individual probe (and therefore each gene is represented by 3 species-specific probe-sets). The array includes a total of 368,678 probes, with 126,763 probes from human, 122,387 from chimpanzee, and 119,528 from rhesus macaque. The percentage of genes having exactly 7 probes is 99.9%, 91.5%, and 85.3% for human, chimpanzee, and rhesus macaque, respectively. In addition, a set of random sequence probes was included on the array as controls. As expected, these probes generally showed low intensity values in all hybridizations.

### Microarray Study Design and Low-Level Analysis

Complete details about the study design, samples used, hybridization procedures, and quality control analyses are given in Text S1 and Tables S1 and S2. Briefly, using the multi-species microarray, we compared gene expression levels within and between species in three tissues: Livers, Kidneys (cortex) and Heart muscle. For each tissue, we hybridized RNA samples from 6 individuals from each of the three species, and preformed two technical replicates for each sample. The total number of arrays analyzed is therefore 108 ( = 3 species×3 tissues×6 individuals×2 technical replicates). Gel pictures of all RNA samples are available in Figure S18.

Following hybridization, washing, and scanning, raw data was extracted from the images using the NimbleScan software (version 2.4). We performed background correction using the normexp function in limma with an offset of 32 [60], and normalization using an adaptation of the quantile normalization approach.

### Statistical Analysis

We used the following gene specific linear mixed model to analyze the background corrected normalized data for each tissue
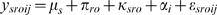
(1)where *y_sroij_* is the normalized log_2_ intensity for species *s* (*s* = human, chimpanzee or rhesus macaque), from individual *i* in replicate *j* from a specific probe within a probe-set *r* which was derived from species *o*. The term *μ_s_* is the expected log expression level of species *s*. The term *π_ro_* represents the probe effect for each individual probe within a probe-set and the effect of species-specific orthologous probes [61]. The *κ_sro_* represent the attenuation on hybridization intensities due to sequence mismatches between species of RNA (*s*) and a species-specific derived probe (*o*), which are different for each individual probe within a probe-set (*r*). We assumed that *κ_sro_* = 0 when *s* is the same species as *o*, and that the attenuation is symmetric for combinations of RNA species and probe ortholog species (i.e., *κ_sro_* = *κ_ors_*). The term *α_i_* is a random effect representing the effect for individuals *i* assumed to be normal with mean zero and variance σ_α_^2^, and *ε_sroij_* is the residual error assumed to be normal with mean zero and variance σ_ε_^2^. The model was fitted to each gene by residual maximum likelihood using the lme function (in the nlme package). We used likelihood ratio (LR) tests within the framework of the linear model in order to identify genes that are differentially expressed (DE) between species (see Text S1 for more details). The reported *P*-values were adjusted for multiple testing using the false discovery rate approach (FDR; [62]).

To identify genes whose regulation likely evolves under *stabilizing* selection in the three primate species, we used two criteria. First, we wanted to exclude genes with evidence for differential expression between species (as such a pattern is not consistent with stabilizing selection on gene expression levels). To so do, we used a likelihood ratio test to test the null hypothesis that there are no expression differences between species (i.e. *μ_H_* = *μ_R_* = *μ_C_*). Under the null hypothesis, −2×(log-likelihood ratio) of the fits of the reduced and full model has an approximate χ^2^ distribution on 2 degrees of freedom. Since our goal at this step is to exclude genes that are DE between species, we retained genes where this statistic was less than 6 (corresponding to *P*>0.05). Among the genes that are *not* DE between species, those whose regulation evolves under stabilizing selection are expected to have low between-individuals variance. Figures 2 and S13 illustrate examples of expression patterns that are consistent with such expectation. Thus, we ranked the remaining genes by their between individuals variance (see Text S1 for more details).

Finally, we excluded genes that had very low expression levels, as these might have low variance within and between species simply because they are not expressed. To do so, we calculated the average normalized log-expression level for each gene across all probes, plotted this intensity against the between-individual variance, and selected a cutoff that excluded genes within the obvious cluster of small absolute intensity values (Figure S14). Using this approach, we excluded genes with log absolute intensity values smaller than 7 for liver (23% of genes excluded), 6.7 for kidney (17% of genes excluded), and 7 for heart (26% of genes excluded).

To find genes whose regulation likely evolved under *directional* selection in humans, we focused on genes whose expression level has changed exclusively in either the human or the chimpanzee lineage, as well as maintained low within-species variance. Figure S15 illustrates examples of expression patterns that are consistent with such expectation. To identify these patterns, we used three criteria: first, we excluded genes that are DE between the non-human primates. To do so, we constructed a reduced model to test if the chimpanzee and rhesus macaque expression levels are similar (i.e., *μ_C_* = *μ_R_*); the maximum likelihood estimate was compared to the full model in [1]. Genes that are differentially expressed between chimpanzee and rhesus macaque will have a high likelihood ratio; therefore we excluded them from further analyses (using a LR test cutoff of 2). Among genes with consistent expression level in the non-human primates, we selected those that have a significantly different expression levels in humans, by using a second LR test. Here, we tested a model that reflects the assumption of similar expression levels in chimpanzee and rhesus macaque (*μ_C_* = *μ_R_*) against a null model that reflects the assumption of similar expression for all species (*μ_H_* = *μ_R_* = *μ_C_*), this time retaining genes for which we could reject the null (using an LR test cutoff of 10). Finally, we ranked these genes by their between individuals variance.

### Analysis for Enrichments of Functional Categories and Pathways

In order to identify functional categories and pathways that are enriched among genes with either high or low between individual variance in gene expression, we applied either a Fisher Exact Test (FET), using 2×2 contingency tables, or a Mann-Whitney test, using ranks (e.g., the rank of the between individual variance). We excluded from this analysis, and the following ‘enrichment’ analyses genes that do not have a record in GO, in order to avoid biasing the results with enriched functional categories that simply have more genes with studied/known functions.

To identify functional categories and pathways that are enriched among genes whose regulation has likely evolved under natural selection, we defined (for each tissue) the following three mutually exclusive gene groups: (i) genes whose regulation has likely evolved under directional selection, (ii) genes whose regulation has likely evolved under stabilizing selection, and (iii) all other genes not in groups (i) or (ii) – referred to as “others” in Table S7. Genes with high between-individual variance were excluded from group (iii) because they can never be included in groups (i) or (ii) (including these genes in group (iii) may bias the results). To test for enrichment we performed a two-tailed FET (using the fisher.test function).

For the GO analysis, we initially only asked for enrichment of transcription factors (GO:0030528) and/or metabolic genes (GO:0008152), where we have a strong prior given previous studies, including our own. We did not ask about any other GO functional category and therefore did not correct the P-values reported in Tables 2 and 3 for multiple tests. Thus, our first step represents a test of explicit hypothesis.

As a second step, we performed a global analysis of enrichment in all GO categories under ‘biological processes’ and ‘molecular function’ using DAVID (http://david.abcc.ncifcrf.gov/). The results of this analysis are provided in Table S7. We note that a global analysis of all GO terms is somewhat difficult to interpret, because many of the functional annotations in GO are not mutually exclusive at any level of the hierarchy, and are often not very informative. That said, it can be seen in Table S7 that many of the top results are GO terms related to gene regulation and metabolic pathways, and in particular when we put together all genes whose regulation is inferred to have likely evolved under directional selection, the two top enriched GO terms are ‘transcription factor binding’ (GO:0008134) and ‘metabolic processes’ (GO:0008152). Thus, the results of the global GO analysis are consistent with our hypothesis that transcription factors and genes in metabolic pathways are enriched among genes whose expression profiles have changed exclusively in the human lineage.

### Electronic Database Information

All expression data files were submitted to the GEO database (http://www.ncbi.nlm.nih.gov/geo/) under provisional series accession number GSE11560.

## Supporting Information

Figure S1 An illustration of the microarray hybridization study design.
(0.05 MB DOC)

Figure S2 Boxplots showing the distributions of the log intensities of the raw data.
(0.07 MB DOC)

Figure S3 Distributions of the log intensities of 108 arrays after normalization.
(0.06 MB DOC)

Figure S4 Density distributions of log intensities of all 108 normalized arrays.
(0.03 MB DOC)

Figure S5 MA plots of normalized data for the technical replicates of liver hybridizations.
(0.07 MB DOC)

Figure S6 MA plots of normalized data for the technical replicates of kidney hybridizations.
(0.07 MB DOC)

Figure S7 MA plots of normalized data for the technical replicates of heart hybridizations.
(0.07 MB DOC)

Figure S8 Distributions of pairwise Pearson correlations between arrays, by category.
(0.04 MB DOC)

Figure S9 Estimates of lineage-specific change in gene expression levels in the liver.
(0.07 MB DOC)

Figure S10 Estimates of lineage-specific change in gene expression levels in the liver.
(0.03 MB DOC)

Figure S11 Correspondence between studies/platforms.
(0.07 MB DOC)

Figure S12 Classifying genes according to between-individual variance.
(0.04 MB DOC)

Figure S13 Examples of expression patterns that are consistent with the action of stabilizing selection.
(0.04 MB DOC)

Figure S14 Excluding lowly-expressed genes.
(0.04 MB DOC)

Figure S15 Examples of expression patterns that are consistent with the action of directional selection.
(0.04 MB DOC)

Figure S16 Comparison of data from chimpanzees across tissues.
(0.04 MB DOC)

Figure S17 Protein evolution and selection on gene regulation.
(0.22 MB DOC)

Figure S18 Gel pictures of the 54 total RNA samples.
(0.08 MB DOC)

Table S1 List of genes on the array.
(6.66 MB TXT)

Table S2 Information on the 54 samples used in the study.
(0.08 MB DOC)

Table S3 Analysis of functional categories. Functional categories (top, shaded) and pathways (bottom, clear) that are enriched among genes with high or low between-individual (BI) variance in gene expression.
(0.06 MB DOC)

Table S4 Analysis of functional categories.
(0.04 MB DOC)

Table S5 A comparison of Dn/Ds distributions between genes whose regulation evolved under different evolutionary pressures.
(0.04 MB DOC)

Table S6 Analysis of chromosomal rearrangements.
(0.03 MB DOC)

Table S7 Analysis of enrichment in functional categories using GO.
(0.12 MB XLS)

Text S1 Supplementary methods.
(0.13 MB DOC)
