# Correction: Gene Regulation in Primates Evolves under Tissue-Specific Selection Pressures

**DOI:** 10.1371/annotation/06105fdb-f0fa-4fe2-88d0-db9f6036509f

**Published:** 2009-03-05

**Authors:** Ran Blekhman, Alicia Oshlack, Adrien E. Chabot, Gordon K. Smyth, Yoav Gilad

The images for Figures 4 and 5 were mistakenly swapped. The titles and legends for both figures are correct, but the image for Figure 4 should be the image for Figure 5, and vice versa.

