# Supplementary material for: Gene Regulation in Primates Evolves under Tissue-Specific Selection Pressures
Source: PLoS Genet. 2008 Nov 21;4(11):e1000271. doi: 10.1371/journal.pgen.1000271 (PMC2581600; doi:10.1371/journal.pgen.1000271)
Supplement: Figure S1 — An illustration of the microarray hybridization study design. (0.05 MB DOC) [file pgen.1000271.s001.doc]

**Figure S1:** An illustration of the microarray hybridization study design.
