# Supplementary material for: Gene Regulation in Primates Evolves under Tissue-Specific Selection Pressures
Source: PLoS Genet. 2008 Nov 21;4(11):e1000271. doi: 10.1371/journal.pgen.1000271 (PMC2581600; doi:10.1371/journal.pgen.1000271)

**Figure S2:** Boxplots showing the distributions of the log intensities of the raw data. Each box corresponds to one of the 108 arrays; each panel displays a single tissue, from top to bottom: liver, kidney, and heart.


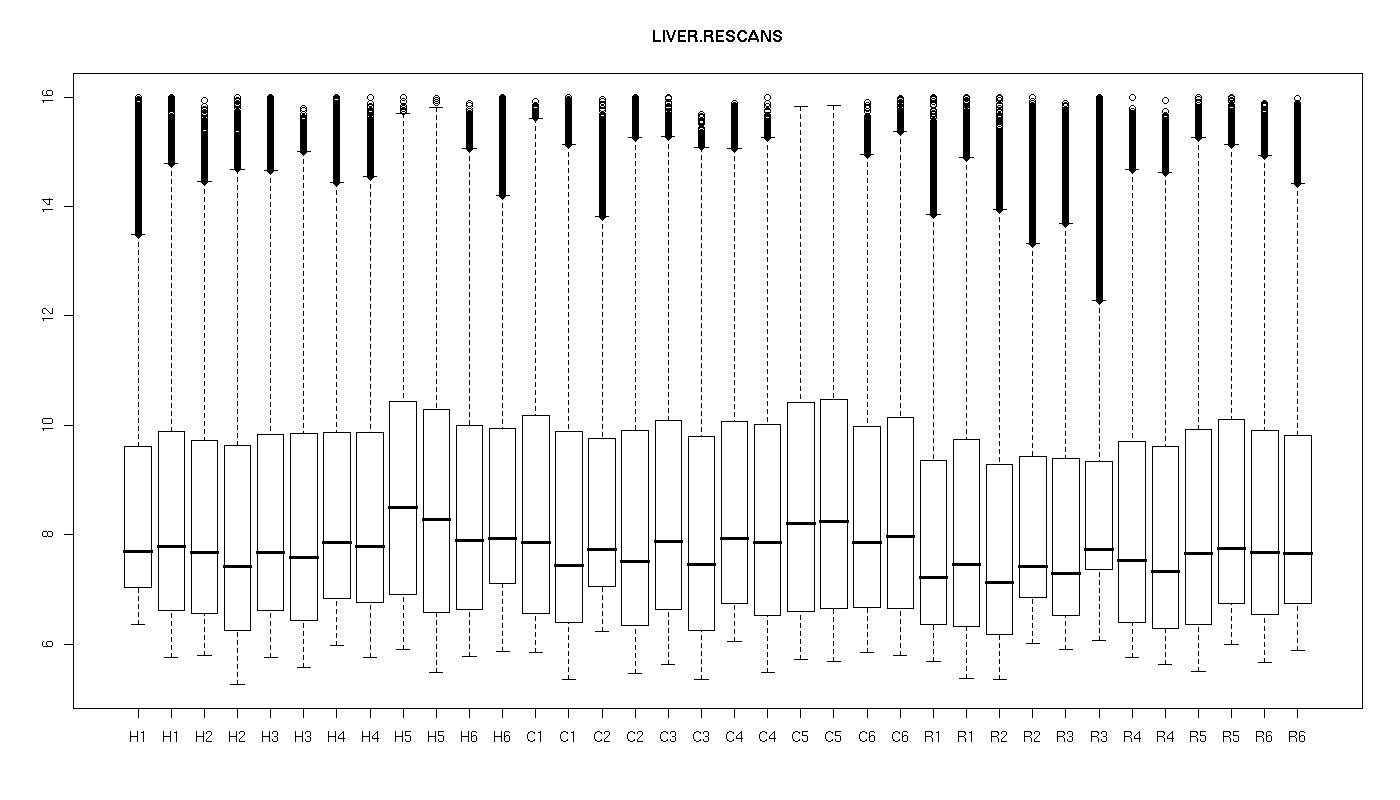


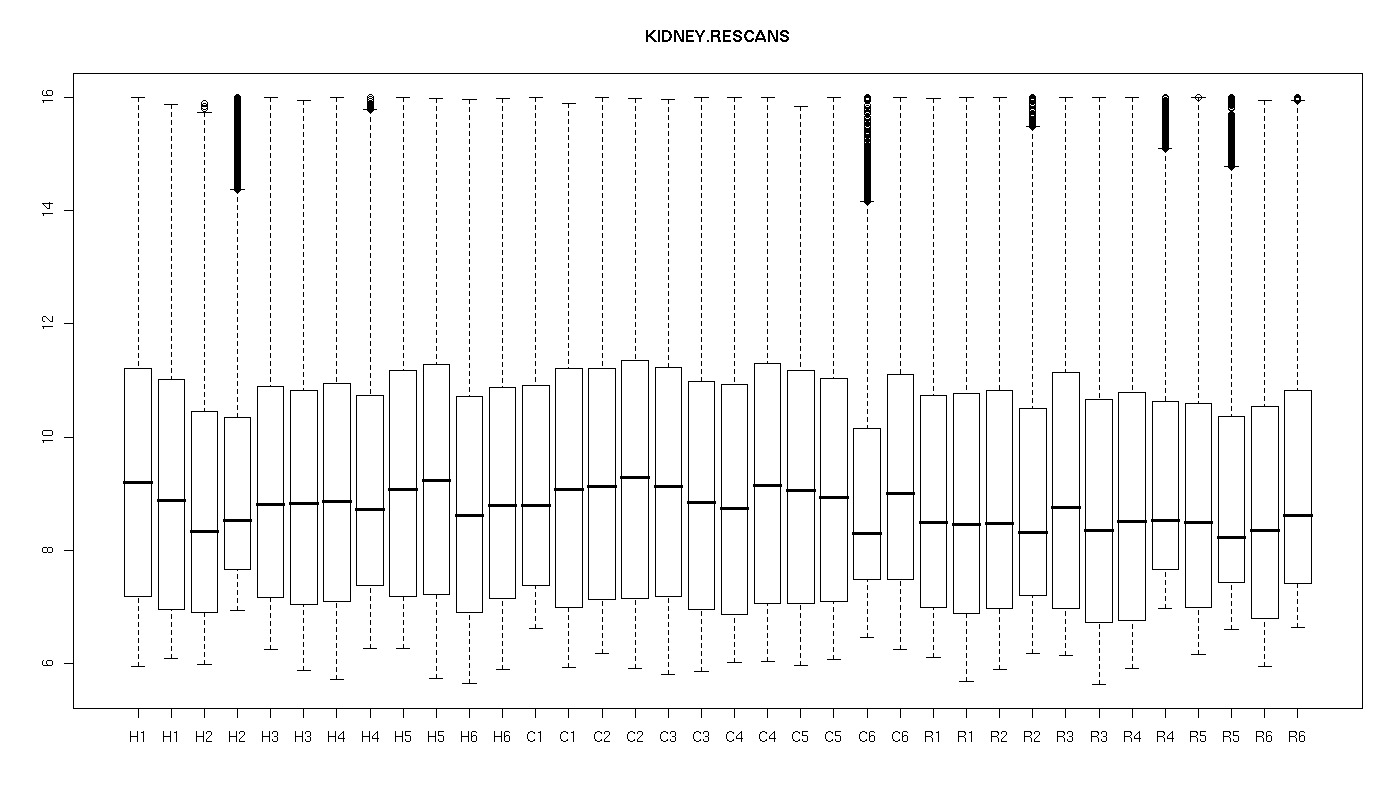


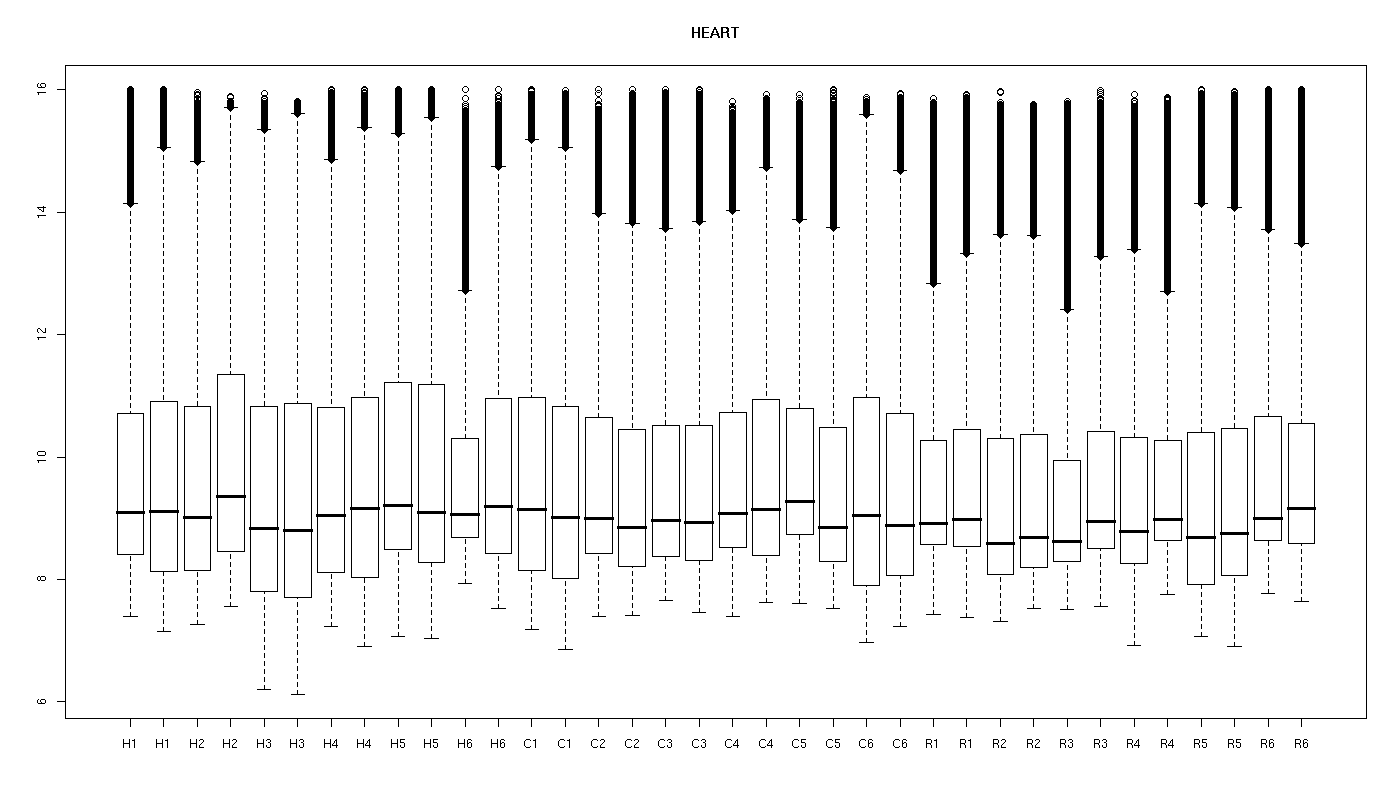

Supplement: Figure S2 — Boxplots showing the distributions of the log intensities of the raw data. (0.07 MB DOC) [file pgen.1000271.s002.doc]
