# Supplementary material for: Gene Regulation in Primates Evolves under Tissue-Specific Selection Pressures
Source: PLoS Genet. 2008 Nov 21;4(11):e1000271. doi: 10.1371/journal.pgen.1000271 (PMC2581600; doi:10.1371/journal.pgen.1000271)

**Figure S3**: Distributions of the log intensities of 108 arrays after normalization. From top to bottom: liver, kidney, and heart.


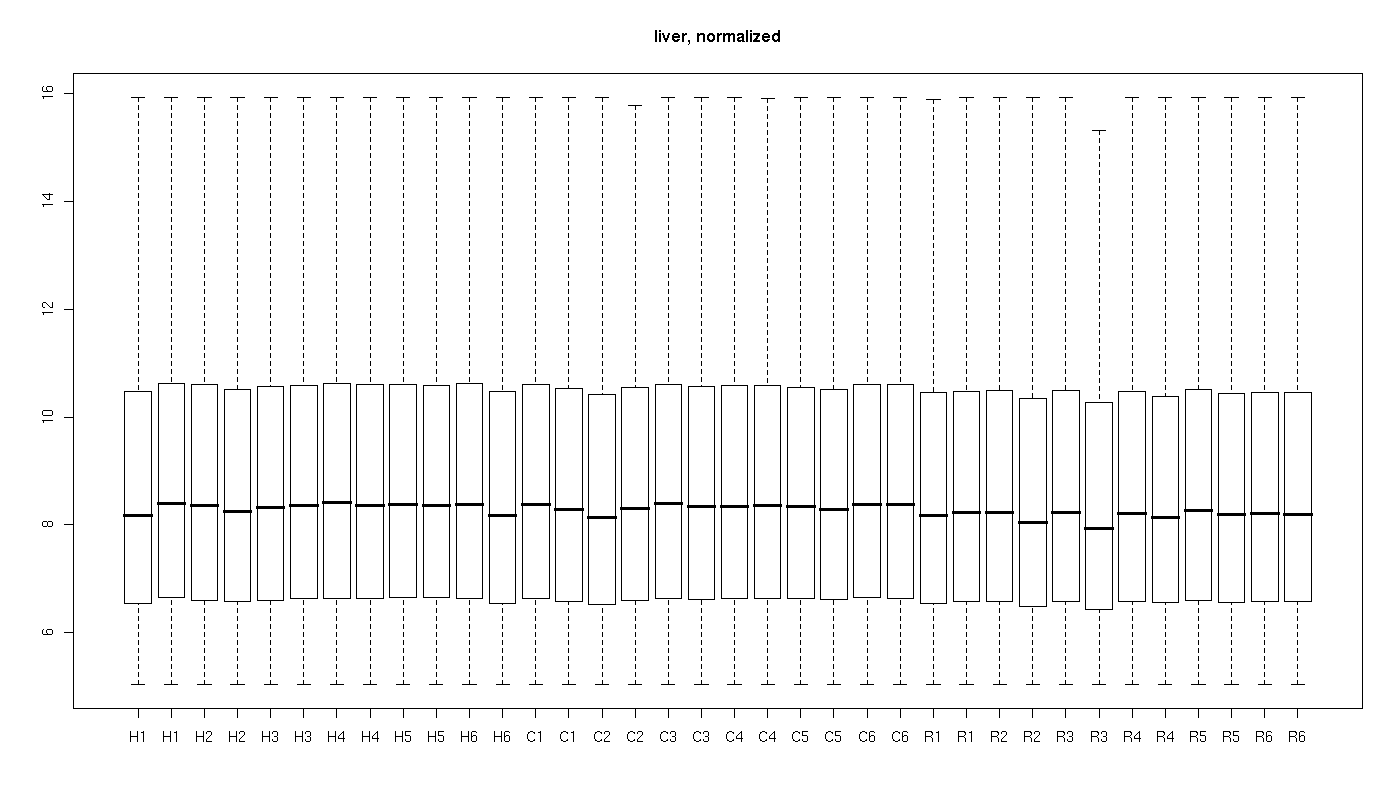


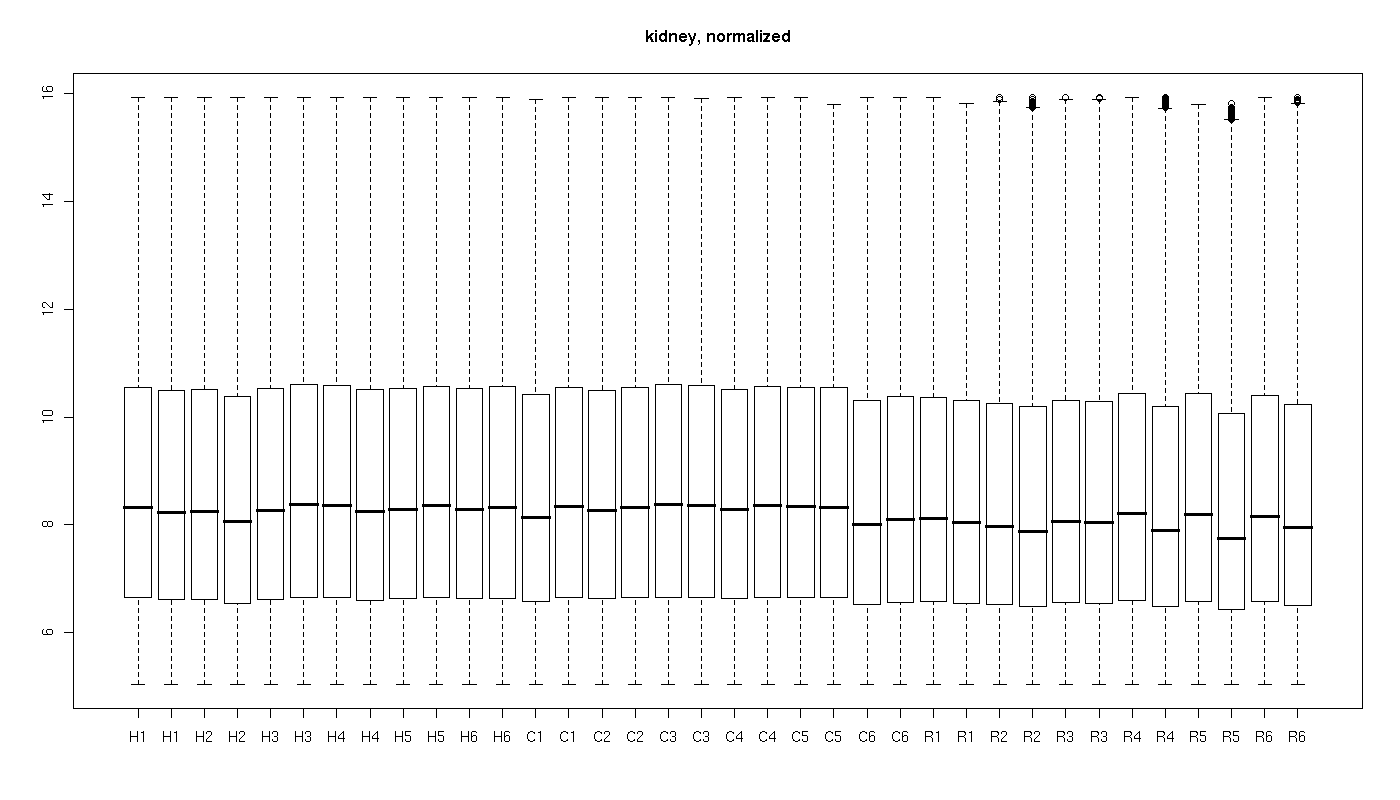


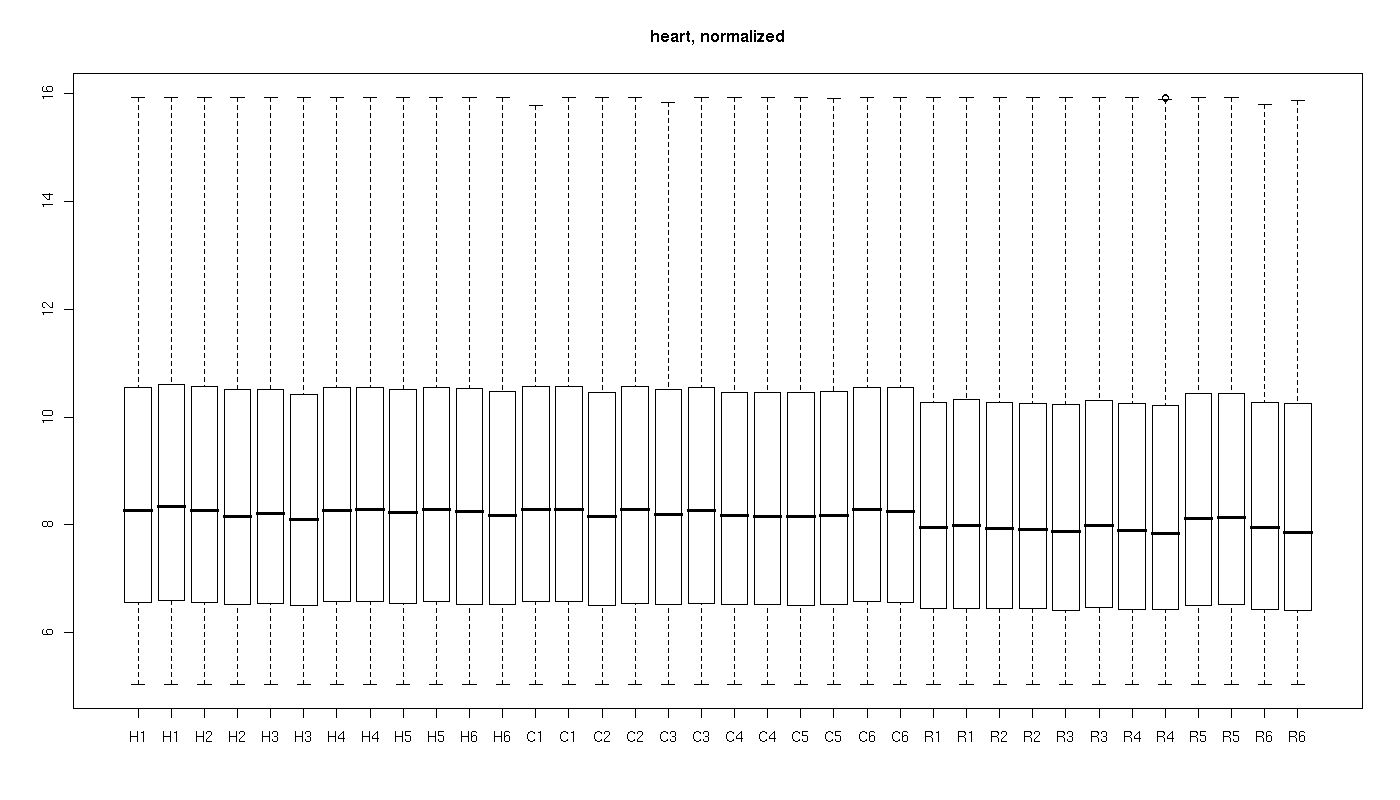

Supplement: Figure S3 — Distributions of the log intensities of 108 arrays after normalization. (0.06 MB DOC) [file pgen.1000271.s003.doc]
