# Supplementary material for: Gene Regulation in Primates Evolves under Tissue-Specific Selection Pressures
Source: PLoS Genet. 2008 Nov 21;4(11):e1000271. doi: 10.1371/journal.pgen.1000271 (PMC2581600; doi:10.1371/journal.pgen.1000271)

**Figure S4:** Density distributions of log intensities of all 108 normalized arrays. Each line represents a single array; red: liver, blue: kidney, green: heart.
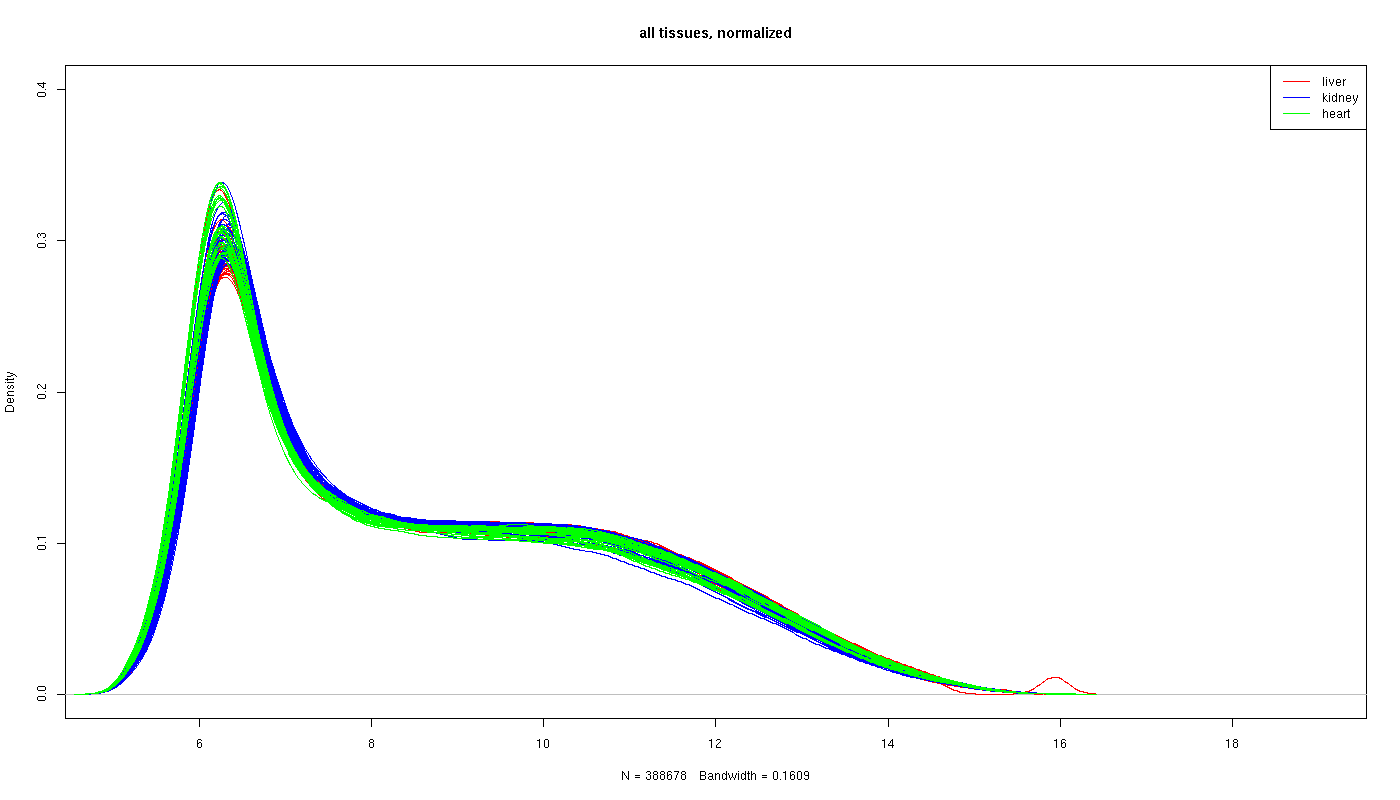

Supplement: Figure S4 — Density distributions of log intensities of all 108 normalized arrays. (0.03 MB DOC) [file pgen.1000271.s004.doc]
