# Supplementary material for: Gene Regulation in Primates Evolves under Tissue-Specific Selection Pressures
Source: PLoS Genet. 2008 Nov 21;4(11):e1000271. doi: 10.1371/journal.pgen.1000271 (PMC2581600; doi:10.1371/journal.pgen.1000271)

**Figure S7**: MA plots of normalized data for the technical replicates of heart hybridizations. Y-axis: M = log_2_(rep1) – log_2_(rep2). X-axis: A = (log_2_(rep1) + log_2_(rep2))/2.


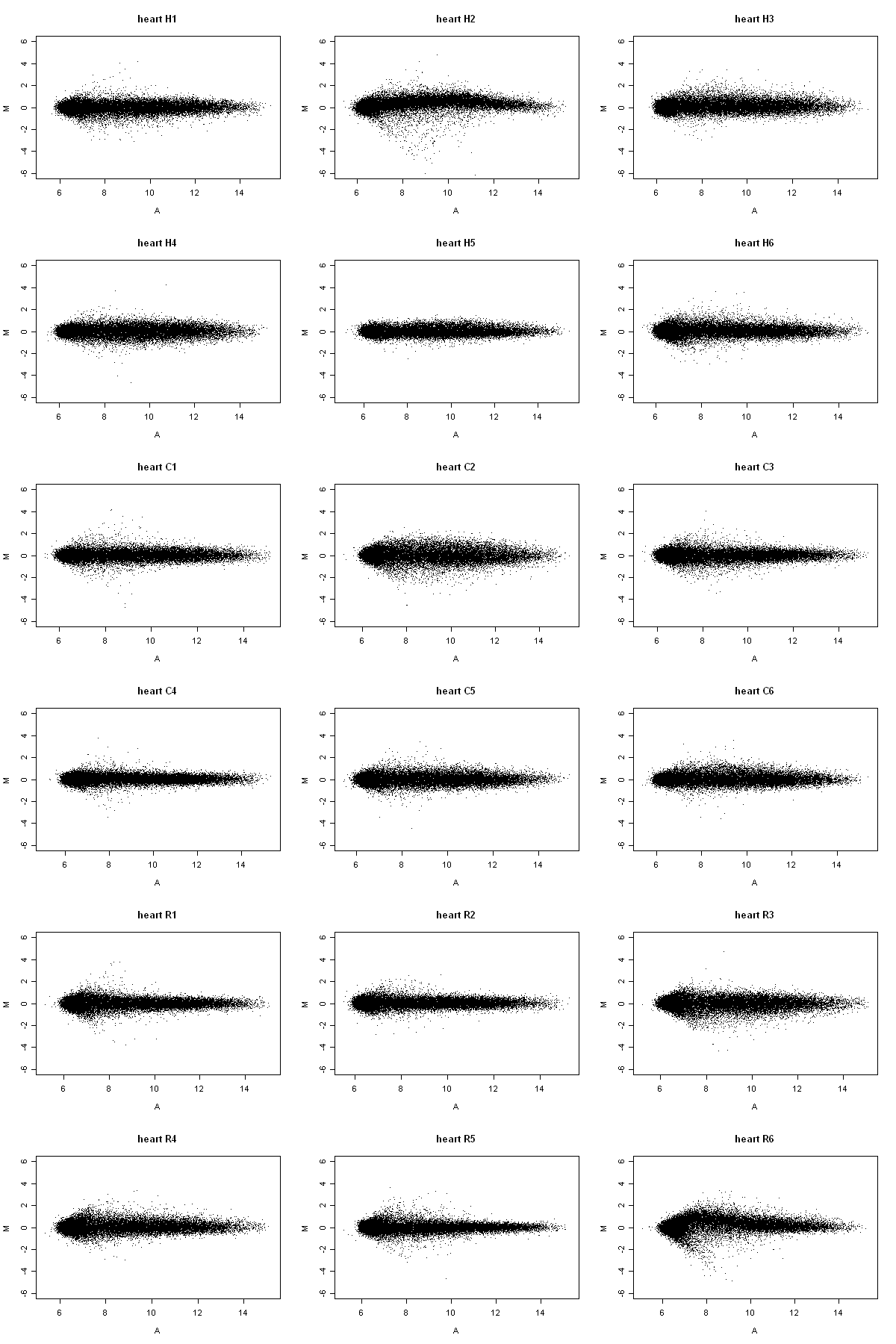

Supplement: Figure S7 — MA plots of normalized data for the technical replicates of heart hybridizations. (0.07 MB DOC) [file pgen.1000271.s007.doc]
