# Supplementary material for: Gene Regulation in Primates Evolves under Tissue-Specific Selection Pressures
Source: PLoS Genet. 2008 Nov 21;4(11):e1000271. doi: 10.1371/journal.pgen.1000271 (PMC2581600; doi:10.1371/journal.pgen.1000271)

**Figure S8**: Distributions of pairwise Pearson correlations between arrays, by category. reps: Technical replicates; wTwS: within tissues, within species (Arrays of same species and tissue excluding technical replicates); wTbS: within tissues between species (Same tissue different species); bTwS: between tissues within species (Same species different tissue); bTbS: between tissues between species (Different species and different tissue).


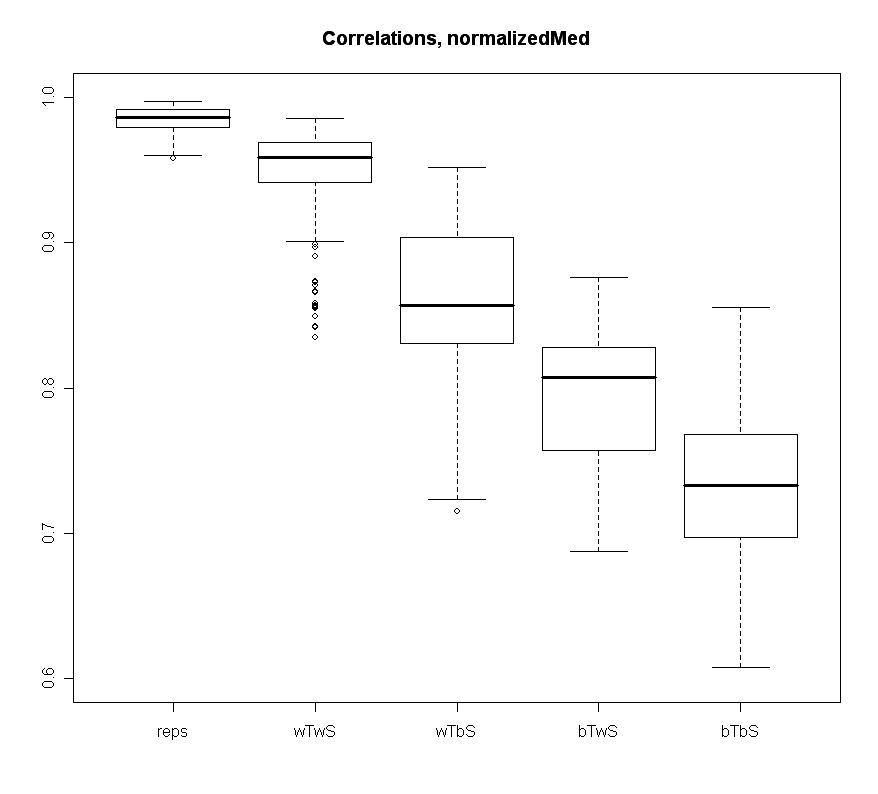

Supplement: Figure S8 — Distributions of pairwise Pearson correlations between arrays, by category. (0.04 MB DOC) [file pgen.1000271.s008.doc]
