# Supplementary material for: Gene Regulation in Primates Evolves under Tissue-Specific Selection Pressures
Source: PLoS Genet. 2008 Nov 21;4(11):e1000271. doi: 10.1371/journal.pgen.1000271 (PMC2581600; doi:10.1371/journal.pgen.1000271)
Supplement: Figure S10 — Estimates of lineage-specific change in gene expression levels in the liver. (0.03 MB DOC) [file pgen.1000271.s010.doc]

**Figure S10**: Estimates of lineage-specific change in gene expression levels in the liver. d_H_ (top) and d_C_ values (bottom), ordered from lowest to highest. Green bars represent positive d values (i.e, expression has increased compared to the rhesus macaque outgroup). Red bars represent negative d values (i.e, expression has decreased compared to the rhesus macaque outgroup)
