# Supplementary material for: Gene Regulation in Primates Evolves under Tissue-Specific Selection Pressures
Source: PLoS Genet. 2008 Nov 21;4(11):e1000271. doi: 10.1371/journal.pgen.1000271 (PMC2581600; doi:10.1371/journal.pgen.1000271)

**Figure S11**: Correspondence between studies/platforms. Log ratios estimated using the pilot cDNA array (x-axis) vs the Nimblegen array (y-axis), using a different set of individuals in each study. The first plot shows chimpanzee to human log ratios and the second plot shows rhesus macaque to human log ratios. Red dot indicate genes which were identified as diferentially expressed in both platforms.

###
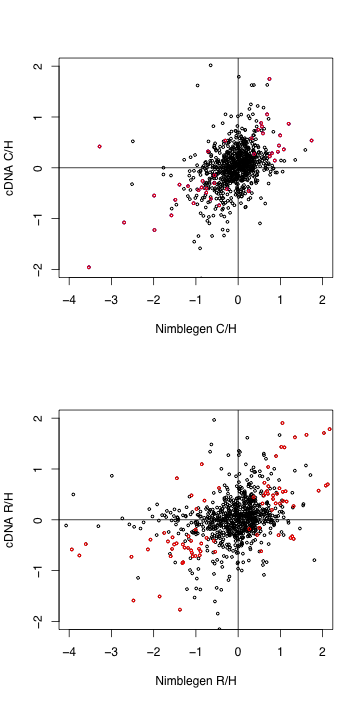

Supplement: Figure S11 — Correspondence between studies/platforms. (0.07 MB DOC) [file pgen.1000271.s011.doc]
