# Supplementary material for: Gene Regulation in Primates Evolves under Tissue-Specific Selection Pressures
Source: PLoS Genet. 2008 Nov 21;4(11):e1000271. doi: 10.1371/journal.pgen.1000271 (PMC2581600; doi:10.1371/journal.pgen.1000271)

**Figure S12**: Classifying genes according to between-individual variance. The distribution of between-individual variance for the three tissues (top to bottom: liver, kidney, and heart). The variance values (y-axis) are plotted against the ranks (x-axis). The dotted line represents the chosen cutoffs, based on a shift in the slope of the distribution (0.4 for liver and kidney, 0.3 for heart).

###
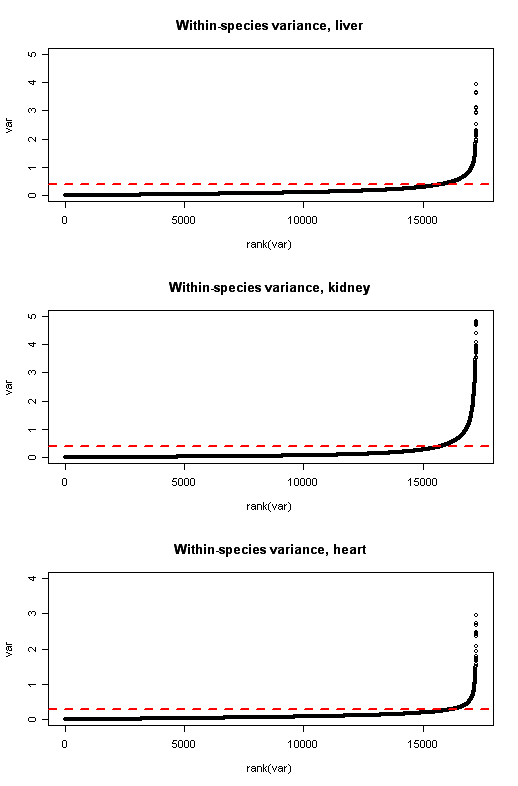

Supplement: Figure S12 — Classifying genes according to between-individual variance. (0.04 MB DOC) [file pgen.1000271.s012.doc]
