# Supplementary material for: Gene Regulation in Primates Evolves under Tissue-Specific Selection Pressures
Source: PLoS Genet. 2008 Nov 21;4(11):e1000271. doi: 10.1371/journal.pgen.1000271 (PMC2581600; doi:10.1371/journal.pgen.1000271)

**Figure S13**: Examples of expression patterns that are consistent with the action of stabilizing selection. Log expression profiles in liver for a sample of six genes whose regulation has likely evolved under stabilizing selection. Each panel illustrates a single gene, where the mean (±s.e.m) log expression level (y-axis) of each species (x-axis) is plotted relative to the human value.


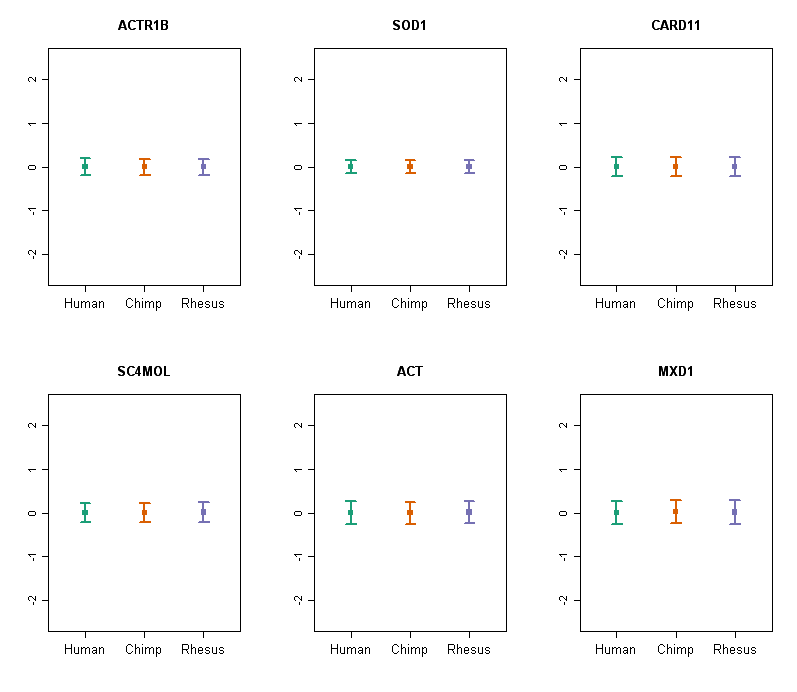

Supplement: Figure S13 — Examples of expression patterns that are consistent with the action of stabilizing selection. (0.04 MB DOC) [file pgen.1000271.s013.doc]
