# Supplementary material for: Gene Regulation in Primates Evolves under Tissue-Specific Selection Pressures
Source: PLoS Genet. 2008 Nov 21;4(11):e1000271. doi: 10.1371/journal.pgen.1000271 (PMC2581600; doi:10.1371/journal.pgen.1000271)

**Figure S14**: Excluding lowly-expressed genes. Average intensity (y-axis) is plotted against estimates of the between-individual variance (x-axis) for each tissue. The broken red line is the cutoff below which genes are likely not to be expressed, hence we excluded these genes from the enrichment analyses.


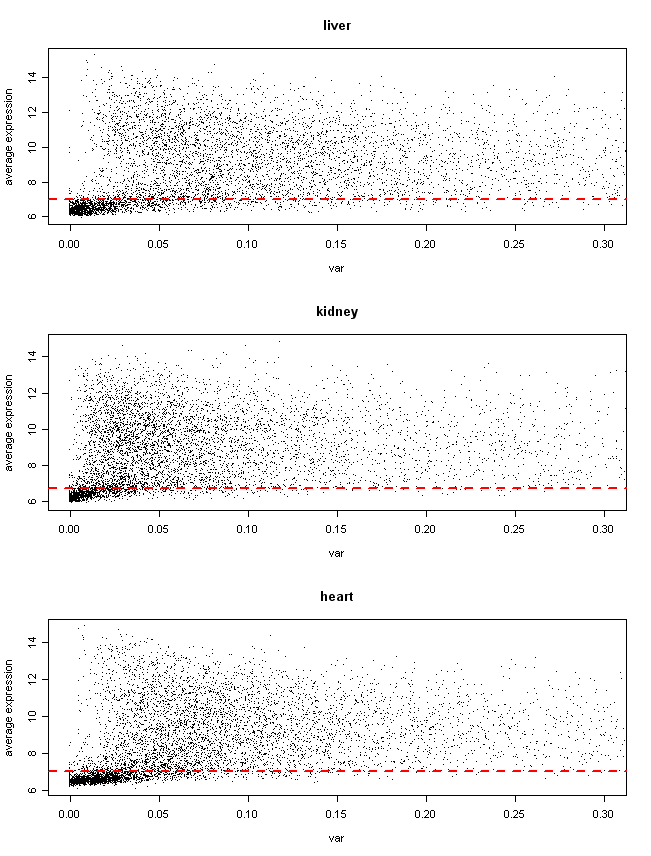

Supplement: Figure S14 — Excluding lowly-expressed genes. (0.04 MB DOC) [file pgen.1000271.s014.doc]
