# Supplementary material for: Gene Regulation in Primates Evolves under Tissue-Specific Selection Pressures
Source: PLoS Genet. 2008 Nov 21;4(11):e1000271. doi: 10.1371/journal.pgen.1000271 (PMC2581600; doi:10.1371/journal.pgen.1000271)

**Figure S15**: Examples of expression patterns that are consistent with the action of directional selection. Log expression profiles in liver for eight genes whose regulation has likely evolved under directional selection in human. Each panel illustrates a single gene, where the mean (±s.e.m) log expression level (y-axis) of each species (x-axis) is plotted relative to the human value. .


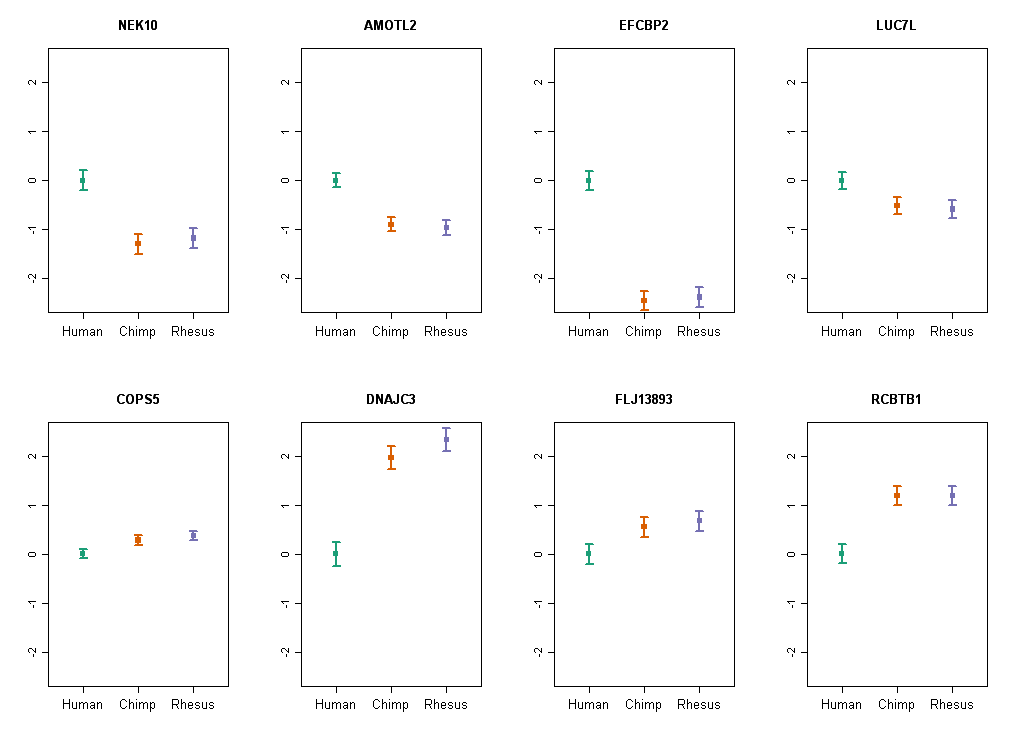

Supplement: Figure S15 — Examples of expression patterns that are consistent with the action of directional selection. (0.04 MB DOC) [file pgen.1000271.s015.doc]
