# Supplementary material for: Gene Regulation in Primates Evolves under Tissue-Specific Selection Pressures
Source: PLoS Genet. 2008 Nov 21;4(11):e1000271. doi: 10.1371/journal.pgen.1000271 (PMC2581600; doi:10.1371/journal.pgen.1000271)

**Figure S16**: Comparison of data from chimpanzees across tissues. Venn diagram showing the number of genes whose regulation evolves under directional selection in chimpanzee.


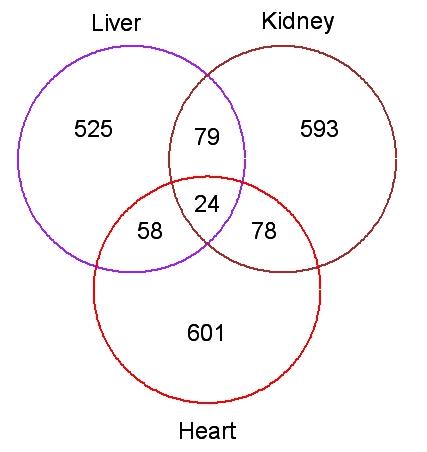

Supplement: Figure S16 — Comparison of data from chimpanzees across tissues. (0.04 MB DOC) [file pgen.1000271.s016.doc]
