# Supplementary material for: Gene Regulation in Primates Evolves under Tissue-Specific Selection Pressures
Source: PLoS Genet. 2008 Nov 21;4(11):e1000271. doi: 10.1371/journal.pgen.1000271 (PMC2581600; doi:10.1371/journal.pgen.1000271)

**Figure S17**: Protein evolution and selection on gene regulation. Cumulative distributions of d_N_/d_S_ values (x-axis) of (**A**) genes whose regulation likely evolved under stabilizing selection in the liver of all three species (red), under stabilizing selection only in rhesus macaque and chimpanzee (green), under directional selection in the human liver (blue), or for which we do not have evidence for selection on gene regulation in the liver (black).

As can be seen from the main paper, genes whose regulation likely evolved under directional selection in humans have low d_N_/d_S_ values. One concern, however, is that such genes were under stabilizing selection for most of the time since the divergence of human and rhesus macaque, and so comparing such genes to those whose regulation evolved under no selection (i.e., the “other” group) may bias the d_N_/d_S_ values downward. While we cannot study directly the selection pressures on gene regulation in the common ancestor of human and chimpanzee, we attempted to address this issue by identifying a fourth group of genes: Those whose regulation likely evolved under stabilizing selection in rhesus macaque and chimpanzee, but not in human. The regulation of genes in the fourth group likely evolved under stabilizing selection since the divergence of human and rhesus macaque, except in the human lineage. As can be seen below, d_N_/d_S_ values for the fourth group are not reduced (results are shown for liver expression data; results were similar regardless of the tissue).


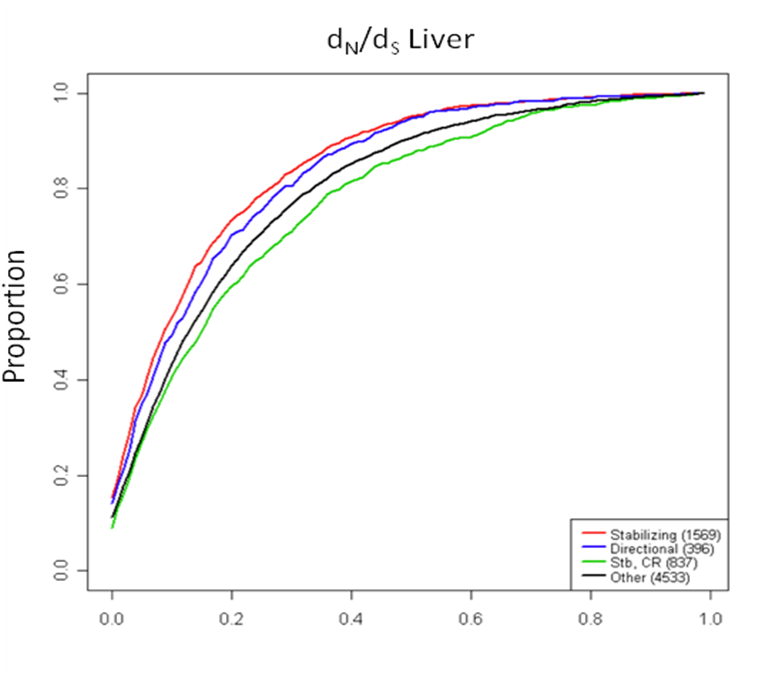

Supplement: Figure S17 — Protein evolution and selection on gene regulation. (0.22 MB DOC) [file pgen.1000271.s017.doc]
