# Supplementary figures and images for: Gene Regulation in Primates Evolves under Tissue-Specific Selection Pressures
Source: PLoS Genet. 2008 Nov 21;4(11):e1000271. doi: 10.1371/journal.pgen.1000271 (PMC2581600; doi:10.1371/journal.pgen.1000271)

**Figure S18**: Gel pictures of the 54 total RNA samples.


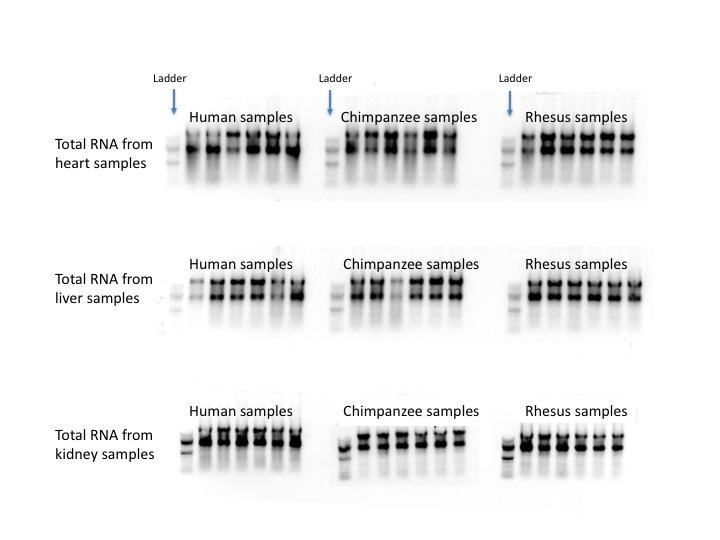

Supplement: Figure S18 — Gel pictures of the 54 total RNA samples. (0.08 MB DOC) [file pgen.1000271.s018.doc]
