# Supplementary material for: Gene Regulation in Primates Evolves under Tissue-Specific Selection Pressures
Source: PLoS Genet. 2008 Nov 21;4(11):e1000271. doi: 10.1371/journal.pgen.1000271 (PMC2581600; doi:10.1371/journal.pgen.1000271)
Supplement: Table S2 — Information on the 54 samples used in the study. (0.08 MB DOC) [file pgen.1000271.s020.doc]

**Table S2:** Information on the 54 samples used in the study.

| **Livers** |  | **Source** | **species** | **ID** | **sex** |
| --- | --- | --- | --- | --- | --- |
|  | H1 | Yale | Human | Yale 1 | m |
|  | H2 | Yale | Human | Yale 2 | m |
|  | H3 | Yale | Human | Yale 3 | m |
|  | H4 | Yale | Human | Yale 4 | m |
|  | H5 | NDRI | Human | 56655 | f |
|  | H6 | NDRI | Human | 56720 | f |
|  | C1 | Yerkes | Chimpanzee | YN05-400, Jeanie | f |
|  | C2 | Yerkes | Chimpanzee | YN06-108, Beleka | f |
|  | C3 | MD Anderson | Chimpanzee | MDANDER | m |
|  | C4 | Yerkes | Chimpanzee | YN06-147, Iyk | m |
|  | C5 | Yerkes | Chimpanzee | c0547, Keith | m |
|  | C6 | A.Stone | Chimpanzee | YN95-427 | m |
|  | R1 | Yerkes | Rhesus macaque | YN05-349, RFj9 | f |
|  | R2 | Yerkes | Rhesus macaque | YN05-82, RFh3 | m |
|  | R3 | A.Stone | Rhesus macaque | 13330-305, R1333 | m |
|  | R4 | A.Stone | Rhesus macaque | 19935-305, R1999 | m |
|  | R5 | Yerkes | Rhesus macaque | YN04-311 | f |
|  | R6 | Yerkes | Rhesus macaque | 17602 | m |

| **Kidneys** |  | **Source** | **species** | **ID** | **sex** |
| --- | --- | --- | --- | --- | --- |
|  | H1 | Kevin Zorn, UoC | Human | KZ001 | m |
|  | H2 | NDRI | Human | 58692 | f |
|  | H3 | NDRI | Human | 56749 | f |
|  | H4 | NDRI | Human | 56859 | f |
|  | H5 | NDRI | Human | 58690 | f |
|  | H6 | NDRI | Human | 58753 | m |
|  | C1 | SFBR | Chimpanzee | 4-0191 | f |
|  | C2 | Varki | Chimpanzee | 0119 | m |
|  | C3 | Yerkes | Chimpanzee | YN05-400, Jeanie | f |
|  | C4 | Yerkes | Chimpanzee | YN06-108, Beleka | f |
|  | C5 | Yerkes | Chimpanzee | YN06-147, Iyk | m |
|  | C6 | MD Anderson | Chimpanzee | MDANDER | m |
|  | R1 | Yerkes | Rhesus macaque | YN05-115, RMe4 | f |
|  | R2 | Yerkes | Rhesus macaque | YN05-349, RFj9 | f |
|  | R3 | Yerkes | Rhesus macaque | YN06-52, RFv7 | m |
|  | R4 | Yerkes | Rhesus macaque | YN06-80, RLh7 | f |
|  | R5 | Yerkes | Rhesus macaque | YN06-82, RFh3 | m |
|  | R6 | SFBR | Rhesus macaque | 17550 | f |

**Table S2 (continued)**

| **Hearts** |  | **Source** | **species** | **ID** | **sex** |
| --- | --- | --- | --- | --- | --- |
|  | H1 | NDRI | Human | 59511 | m |
|  | H2 | NDRI | Human | 58937 | m |
|  | H3 | NDRI | Human | 59263 | m |
|  | H4 | NDRI | Human | 59167 | m |
|  | H5 | NDRI | Human | 59365 | m |
|  | H6 | NDRI | Human | 59303 | m |
|  | C1 | Yerkes | Chimpanzee | YN06-147 | m |
|  | C2 | SFBR | Chimpanzee | c0563 | m |
|  | C3 | MD Anderson | Chimpanzee | MDANDER | m |
|  | C4 | SFBR | Chimpanzee | 4x0519 | m |
|  | C5 | SFBR | Chimpanzee | 4x0516 | m |
|  | C6 | Yerkes | Chimpanzee | Duncan | m |
|  | R1 | Yerkes | Rhesus macaque | YN06-297 | m |
|  | R2 | Yerkes | Rhesus macaque | YN06-300 | m |
|  | R3 | SFBR | Rhesus macaque | 18405 | m |
|  | R4 | Yerkes | Rhesus macaque | YN07-37 | f |
|  | R5 | Yerkes | Rhesus macaque | YN06-295 | m |
|  | R6 | Yerkes | Rhesus macaque | YN06-259 | f |
