# Supplementary material for: Gene Regulation in Primates Evolves under Tissue-Specific Selection Pressures
Source: PLoS Genet. 2008 Nov 21;4(11):e1000271. doi: 10.1371/journal.pgen.1000271 (PMC2581600; doi:10.1371/journal.pgen.1000271)
Supplement: Table S3 — Analysis of functional categories. Functional categories (top, shaded) and pathways (bottom, clear) that are enriched among genes with high or low between-individual (BI) variance in gene expression. (0.06 MB DOC) [file pgen.1000271.s021.doc]

**Table S3:** Analysis of functional categories**.** Functional categories (top, shaded) and pathways (bottom, clear) that are enriched among genes with high or low between-individual (BI) variance in gene expression.

|  | **Tissue** | **Category** | ***P*-value** |
| --- | --- | --- | --- |
| **High BI Variance** | Liver | Metabolic disease | < 10^-7^ |
|  |  | Essential | < 10^-5^ |
|  |  | Obesity | < 10^-5^ |
|  |  | Dyslipidemia | < 10^-3^ |
|  |  | Cytokine-cytokine receptor interaction | < 10^-10^ |
|  |  | Cell adhesion molecules (CAMs) | < 10^-8^ |
|  |  | Leukocyte transendothelial migration | < 10^-5^ |
|  |  | Natural killer cell mediated cytotoxicity | < 10^-5^ |
|  |  | Neuroactive ligand-receptor interaction | < 10^-5^ |
|  | Kidney | OMIM | < 10^-21^ |
|  |  | Hypertension | < 10^-10^ |
|  |  | Dyslipidemia | < 10^-7^ |
|  |  | Obesity | < 10^-7^ |
|  |  | Metabolism of xenobiotics by cytochrome P450 | < 10^-14^ |
|  |  | PPAR signaling pathway | < 10^-11^ |
|  |  | Complement and coagulation cascades | < 10^-10^ |
|  |  | Pentose and glucuronate interconversions | < 10^-10^ |
|  |  | Androgen and estrogen metabolism | < 10^-8^ |
|  | Heart | Metabolic disease | < 10^-6^ |
|  |  | Obesity | < 10^-6^ |
|  |  | Essential | < 10^-3^ |
|  |  | T2D | 0.004 |
|  |  | Complement and coagulation cascades | < 10^-4^ |
|  |  | Cell Communication | < 10^-4^ |
|  |  | Cell adhesion molecules (CAMs) | < 10^-4^ |
|  |  | Neuroactive ligand-receptor interaction | < 10^-4^ |
|  |  | Linoleic acid metabolism | < 10^-3^ |
| **Low BI Variance** | Liver | Housekeeping | < 10^-15^ |
|  |  | Metabolic (GO) | < 10^-9^ |
|  |  | Transcription factors (Validated) | 0.001 |
|  |  | Ribosome | 0.001 |
|  |  | Oxidative phosphorylation | 0.001 |
|  | Kidney | Housekeeping | < 10^-18^ |
|  |  | Transcription factors (Validated) | < 10^-7^ |
|  |  | Metabolic (GO) | < 10^-4^ |
|  |  | Ribosome | < 10^-3^ |
|  |  | Oxidative phosphorylation | < 10^-3^ |
|  | Heart | Housekeeping | < 10^-13^ |
|  |  | Metabolic (GO) | < 10^-6^ |
|  |  | Transcription factors (Validated) | < 10^-3^ |
|  |  | Cytokine-cytokine receptor interaction | < 10^-3^ |
