# Supplementary material for: Gene Regulation in Primates Evolves under Tissue-Specific Selection Pressures
Source: PLoS Genet. 2008 Nov 21;4(11):e1000271. doi: 10.1371/journal.pgen.1000271 (PMC2581600; doi:10.1371/journal.pgen.1000271)
Supplement: Table S4 — Analysis of functional categories. (0.04 MB DOC) [file pgen.1000271.s022.doc]

**Table S4:** Analysis of functional categories. Functional categories (top, shaded) and pathways (bottom, clear) that are enriched among genes whose regulation likely evolves under directional selection in chimpanzee.

| **Tissue** | **Category** | ***P*-value** | Comment |
| --- | --- | --- | --- |
| **Liver** | Associated with cancer | 0.025 | Under-represented |
|  | Transcription factors (GO) | 0.041 | Under-represented |
|  | PPAR signaling pathway | 0.006 |  |
|  | Ubiquitin mediated proteolysis | 0.016 |  |
|  | Atrazine degradation | 0.019 |  |
|  | Focal adhesion | 0.027 |  |
|  | Taurine and hypotaurine metabolism | 0.030 |  |
| **Kidney** | Associated with cancer | 0.006 |  |
|  | Ascorbate and aldarate metabolism | 0.007 |  |
|  | Huntington's disease | 0.009 |  |
|  | Fructose and mannose metabolism | 0.010 |  |
|  | Lysine degradation | 0.012 |  |
|  | beta-Alanine metabolism | 0.015 |  |
| **Heart** | Metabolic (GO) | 0.002 |  |
|  | Housekeeping | 0.016 |  |
|  | Vitamin B6 metabolism | 0.019 |  |
|  | Proteasome | 0.045 |  |
|  | Folate biosynthesis | 0.049 |  |
