# Supplementary material for: Gene Regulation in Primates Evolves under Tissue-Specific Selection Pressures
Source: PLoS Genet. 2008 Nov 21;4(11):e1000271. doi: 10.1371/journal.pgen.1000271 (PMC2581600; doi:10.1371/journal.pgen.1000271)
Supplement: Table S5 — A comparison of Dn/Ds distributions between genes whose regulation evolved under different evolutionary pressures. (0.04 MB DOC) [file pgen.1000271.s023.doc]

**Table S5:** A comparison of Dn/Ds distributions between genes whose regulation evolved under different evolutionary pressures. Genes whose regulation evolves under stabilizing or directional selections are grouped under ‘stabilizing’ and ‘directional’, respectively. The ‘other’ notation refers to genes for which we did not find evidence for the action of natural selection on their tissue-specific expression levels. Permutation *P*-values were calculated for a difference in medians using a permutation test. KS *P*-values were calculated using a Kolmogorov-Smirnov test

|  | **Contrast** | **#genes** | **Dn/Ds Medians** | **KS *P*-value** | **Permutation *P*-value** |
| --- | --- | --- | --- | --- | --- |
| **Liver** | Stabilizing vs. Directional | 1578, 401 | 0.097, 0.115 | 0.157 | 0.072 |
|  | Stabilizing vs. Other | 1578, 5494 | 0.097, 0.147 | < 10^-15^ | < 10^-4^ |
|  | Directional vs. Other | 401, 5494 | 0.115, 0.147 | 0.006 | 0.005 |
| **Kidney** | Stabilizing vs. Directional | 1467, 372 | 0.102, 0.120 | 0.156 | 0.039 |
|  | Stabilizing vs. Other | 1467, 5634 | 0.102, 0.144 | < 10^-11^ | < 10^-4^ |
|  | Directional vs. Other | 372, 5634 | 0.120, 0.144 | 0.024 | 0.022 |
| **Heart** | Stabilizing vs. Directional | 1389.,484 | 0.095, 0.110 | 0.0645 | 0.037 |
|  | Stabilizing vs. Other | 1389, 5600 | 0.095, 0.145 | < 10^-15^ | < 10^-4^ |
|  | Directional vs. Other | 484 ,5600 | 0.110, 0.145 | 8.80E-05 | < 10^-4^ |
| **Stabilizing selection on gene regulation** | 3 tissues vs. 2 tissues | 197, 940 | 0.084, 0.096 | 0.135 | 0.137 |
|  | 3 tissues vs. 1 tissue | 197, 1963 | 0.084, 0.107 | 0.023 | 0.024 |
|  | 2 tissues vs. 1 tissue | 940, 1963 | 0.096, 0.107 | 0.038 | 0.036 |
