# Supplementary material for: Gene Regulation in Primates Evolves under Tissue-Specific Selection Pressures
Source: PLoS Genet. 2008 Nov 21;4(11):e1000271. doi: 10.1371/journal.pgen.1000271 (PMC2581600; doi:10.1371/journal.pgen.1000271)
Supplement: Table S6 — Analysis of chromosomal rearrangements. (0.03 MB DOC) [file pgen.1000271.s024.doc]

**Table S6:** Analysis of chromosomal rearrangements. Chromosomal start and end positions (given in hg18 coordinates) of the eight large-scale rearrangements used in our analysis. The table was taken from Marques-Bonet *et al.*, (Genome Biology, 2007), where the original positions were given in hg16 coordinates.

| Chromosome | Inversion start | Inversion end |
| --- | --- | --- |
| 2 | 113968415 | 114077148 |
| 4 | 44509907 | 86221993 |
| 5 | 18434022 | 96023456 |
| 9 | 46992408 | 88161650 |
| 12 | 20833487 | 66695639 |
| 16 | 34030652 | 45066095 |
| 17 | 7868374 | 45104642 |
| 18 | 5961 | 16900536 |
