# Supplementary material for: Gene Regulation in Primates Evolves under Tissue-Specific Selection Pressures
Source: PLoS Genet. 2008 Nov 21;4(11):e1000271. doi: 10.1371/journal.pgen.1000271 (PMC2581600; doi:10.1371/journal.pgen.1000271)
Supplement: Text S1 — Supplementary methods. (0.13 MB DOC) [file pgen.1000271.s026.doc]

**Gene regulation in primates evolves under tissue-specific selection pressures**

Ran Blekhman^1*^, Alicia Oshlack^2*^, Adrien E. Chabot^1^, Gordon K. Smyth^2^ and Yoav Gilad^1^

^1^ Department of Human Genetics, University of Chicago, Chicago, IL 60637

^2^ Walter and Eliza Hall Institute of Medical Research, Parkville, Vic 3050, Australia

^*^These authors contributed equally to this work

Correspondence should be addressed to Yoav Gilad ([gilad@uchicago.edu](mailto:gilad@uchicago.edu))

## Supplementary methods

In the following, we repeat some of the text from the Method section of the main paper. We chose to provide those sections here in order to have a complete, uninterrupted description of the study design and statistical analysis in one file.

**Multi-species array design**

All known human mRNA sequences were downloaded from the RefSeq database (www.ncbi.nih.gov/RefSeq) in August 2006 (RefSeq release 18). When multiple variants existed for the same gene, we considered only the longest available transcript. To find the non-human primate orthologous sequences for the human mRNAs, we downloaded the full genome sequences of chimpanzee (*Pan troglodytes*, March 2006 draft, panTro2) and rhesus macaque (*Macaca mulatta*, January 2006 draft, rheMac2) from the UCSC Genome Browser database ([www.genome.ucsc.edu](http://www.genome.ucsc.edu/)). We then used BLAT [1], to align the human mRNA sequences to the chimpanzee and rhesus macaque genomes. The BLAT algorithm allows aligning mRNA in blocks (corresponding to exons in this case). After filtering the matches by aligned sequence length (the numbers of “matching” aligned bases), we found chimpanzee and rhesus macaque orthologs for 18,109 human genes (complete 3-way alignments are available by request). In our dataset, the average human-chimpanzee percent identity is 99.1%, and the average human-rhesus macaque percent identity is 96.2%. These numbers correspond well with genome-wide averages [2,3]

As a quality control for our alignment, we used a set of high quality human-chimpanzee-rhesus macaque 3-way alignments for 8336 genes, that were published with the rhesus macaque genome paper [3]. For every gene shared in both datasets, we used Blast2Seq [4] to compare the non-human primate sequences across both alignments. The average percent identity between the chimpanzee sequences in the two datasets is 99.93%, where 76% of the sequences are 100% identical. The average percent identity between the rhesus macaque sequences in the two datasets is 99.84%, where 61% of the sequences were 100% identical.

Based on our alignments, probes for the microarray were designed by NimbleGen ([www.nimblegen.com](http://www.nimblegen.com/)). Within each gene, a set of up to 7 non-overlapping 60mer genic regions were chosen as probes from the human gene sequence (hereafter: a probe-set). The corresponding orthologous sequences in the other two genomes defined species-specific probes for chimpanzee and rhesus macaque. Hence each probe-set consists of up to 7 species-specific probes that are aligned to different locations in the gene, and there are 3 species-specific versions for each individual probe (and therefore each gene is represented by 3 species-specific probe-sets). The array includes a total of 368,678 probes, with 126,763 probes from human, 122,387 from chimpanzee, and 119,528 from rhesus macaque. The percentage of genes having exactly 7 probes is 99.9%, 91.5%, and 85.3% for human, chimpanzee, and rhesus macaque, respectively. In addition, a set of random sequence probes was included on the array as controls. As expected, these probes generally showed low intensity values in all hybridizations.

**Sample collection, study design, and array hybridizations**

Using the multi-species microarray, we compared gene expression levels within and between species in three tissues: Livers, kidneys (cortex) and heart muscle. One of the advantages of working with these tissues is that they are relatively homogeneous with respect to cellular composition (e.g., reference [5]). This is in contrast to brain tissue for example, which may differ substantially in cellular composition between samples and, in particular, between species (e.g., reference [6]). As a result, it is unlikely that liver, kidney cortex, or hearts muscle tissue samples from different individuals will be significantly different in terms of cellular composition. In particular, it is unlikely that systematic differences in cellular composition will confound our inter-species analyses.

Non-human livers, kidneys, and hearts were collected for us by the Yerkes primate center, the Southwest Foundation for Biomedical Research, and MD Anderson Cancer Center. Additional primate tissues were given to us by Anne Stone. In all cases, we collected tissues from adult chimpanzees and rhesus macaques that died of natural causes (such as accidents or fights) or were euthanized due to an illness unrelated to liver, kidney, or heart. The human adult tissue samples were collected for us by the National Disease Research Interchange (NDRI), as well as by the pathology departments at the University of Chicago hospital and Yale University (with IRB approval). Detailed information about all samples is available in Table S2.

We extracted RNA from each tissue sample using Trizol (Invitrogen, Carisbad, CA), and synthesized first strand cDNA using a T7-poly-T oligo and the superscript kit (Invitrogen, Carisbad, CA). The labeling and microarray hybridizations of all the liver and kidney samples were performed by Nimblegen, while hybridizations of all the heart samples were performed in the Gilad laboratory, following an identical protocol.

The hybridization study design is illustrated in Figure S1: For each tissue, we hybridized RNA samples from 6 individuals from each of the three species, and preformed two technical replicates for each sample. The total number of arrays analyzed is therefore 108 (= 3 species × 3 tissues × 6 individuals × 2 technical replicates).

Following hybridization, washing, and scanning, raw data was extracted from the images using the NimbleScan software (version 2.4). Figure S2 shows the distributions of the raw data across the 108 arrays.

### Background correction and normalization

All the statistical analyses detailed in this and the following sections were performed in R ([http://www.r-project.org](http://www.r-project.org/)). As a first step, we performed background correction using the normexp method in limma with an offset of 32 [7]. Next, in order to normalize the data, we used an adaptation of the quantile normalization approach. Specifically, we used *only* the distribution of probes corresponding to the hybridization species of each array (i.e., the probes that are not attenuated by sequence mismatches – we often refer to these as the ‘species-specific probes’), as well as the random-sequence probes, to normalize *all* other probes. Thus, as a first step of this normalization approach we determined the quantile distribution from the human specific and random probes for the human hybridizations, the chimpanzee specific and random probes for the chimpanzee hybridizations and the rhesus macaque specific and random probes for the rhesus macaque hybridizations. As the number of probes corresponding to each species differ slightly we interpolated the values for the probes from the larger sets onto the quantile distribution. Next, *all* the probes from each array were normalized to the quantile distribution by fitting the species-specific probes to the empirical distribution, and interpolating the values for probes from the other two species onto the distribution, while maintaining their original ranks. The distribution of raw intensity from all arrays, post-normalization, can be seen in Figures S3 and S4.

### Assessment of array quality and normalization

As well as looking at boxplots of the intensity distribution, we assessed the quality of the raw data by looking at MA plots between replicate arrays (where the x-axis plots A: the average log intensity of a probe/gene across arrays and the y-axis plots M: the log ratio of the probe/gene intensities across arrays). We generated MA plots before and after normalization. Ideally, data should lie on the M=0 axis and deviations indicate variation in probe performance across replicates or ineffective normalization (in particular, when curvatures or offsets are seen in post-normalization data). MA plots of technical replicates (post normalization) are shown in Figures S5-S7, where we have summarized results for each gene by its median value over the probe-set for each species.

An additional (albeit rough) measure of quality is the correlation structure seen in the data (Figure S8). As expected, the correlations between technical replicates are highest (median correlation, r = 0.98). Correlations between other classes are lower, following our intuitive expectation: Correlations between arrays of the same species and tissue (median r = 0.95), is higher than the correlations between arrays of different species in the same tissue (median r = 0.85), which in turn are higher than those of same species in different tissues (median r = 0.8), and finally, the correlations of arrays of different species and tissue is lowest (median r = 0.73).

**Statistical analysis**

For all the following analyses we excluded probes that did not have corresponding orthologs in all three species (i.e., we only consider probes that have the human, chimpanzee, and rhesus macaque species-specific versions on the array – we refer to these as the “corresponding orthologous probes”). We then excluded genes that were represented by fewer than three corresponding orthologous probes across all species. Thus, the total number of genes included in all subsequent analyses was 17,231 (95% of genes originally included on the array). We analyzed data from each tissue (36 arrays) separately to allow for differences in the within-species variance in gene expression across tissues. The number of observations for each gene is 108n, where *n* is the number of corresponding orthologous probes per gene. In other words, for each gene we have information from 36 arrays, *n* corresponding orthologous probes (3 ≤ *n* ≤ 7) per gene, each comprised of 3 species-specific orthologous probes. Thus, we have 36×7×3 = 756 data points from each tissue for genes that are represented by 7 corresponding orthologous probes.

We used the following gene specific linear mixed model to analyze the data for each tissue:

$y_{sroij}=\mu_{s}+\pi_{ro}+\kappa_{sro}+\alpha_{i}+\varepsilon_{sroij}$ [1]

where *y_sroij_* is the normalized log_2_ intensity for species *s* (*s*=human, chimpanzee or rhesus macaque), from individual *i* in replicate *j* from a specific probe within a probe-set *r (r=1…n)* which was derived from species *o* (*o*=human, chimpanzee or rhesus macaque). The term *μ_s_* is the expected log expression level of species *s*. The term *π_ro_* represents the probe effect for each individual probe within a probe-set and the effect of species-specific orthologous probes [8]. The *κ_sro_* represent the attenuation on hybridization intensities due to sequence mismatches between species of RNA (*s*) and a species-specific derived probe (*o*), which are different for each individual probe within a probe-set (*r*). We assume here that *κ_sro_*=0 when *s* is the same species as *o,* and that the attenuation is symmetric for combinations of RNA species and probe orthologous species (i.e. *κ_sro_* = *κ_ors_*). The term *α_i_* is a random effect representing the effect for individuals *i* assumed to be normally distributed with mean zero and variance σ_α_^2^, and *ε_sroij_* is the residual error assumed to be normally distributed with mean zero and variance σ_ε_^2^. The model was fitted to each gene by residual maximum likelihood using the lme function (in the nlme package).

**Identifying differentially expressed genes**

To identify genes that are differentially expressed (DE) *between species within a tissue*, we used likelihood ratio (LR) tests. Specifically, we estimated the maximum likelihood of the full model as well as that of a reduced model, testing the null hypothesis that expression levels between species are the same (e.g., when comparing human and chimpanzee, we constructed a reduced model where *μ_h_*=*μ_c_*). We then calculated −2∙(log-likelihood ratio) between the fits of the reduced and full models. We expect genes that deviate from the null (i.e., genes that are truly differentially expressed between the two species) to have higher values of this statistic. Thus, genes with a likelihood ratio statistic of 10 or higher were selected as differentially expressed between species (this cutoff corresponds to an FDR < 0.01 in all reported comparisons). All *P*-values were calculated under the assumption that the LR has a χ^2^ distribution on one degree of freedom, and then corrected for multiple testing using the false discovery rate procedure [9].

To find genes that are differentially expressed *between tissues within a species*, we used the fitted effects of the tissue-specific linear models and calculated the following z-statistic:

$z=\frac{\mu_{st_{1}}-\mu_{st_{2}}}{\sqrt{SE_{st_{1}}^{2}+SE_{st_{2}}^{2}}}$ [2]

where *s* denotes the species of interest and *t_1_* and *t_2_* are the tissues compared. $\mu_{st_{1}}$ and $\mu_{st_{2}}$ denote the estimated log expression levels for species *s* in tissues *t_1_* and *t_2_*, respectively. $SE_{st_{1}}$ and $SE_{st_{2}}$ denote the standard errors associated with expression level$\mu_{s}$ in tissues *t_1_* and *t_2_*, respectively. Under the null hypothesis, the statistic has a normal distribution with mean 0 and variance 1. *P*-values that correspond to the values of this statistic were calculated and adjusted for multiple testing using the false discovery rate procedure. We used a cutoff of an FDR < 0.01 to identify DE genes between tissues.

**Lineage-specific changes in gene expression**

In order to infer lineage-specific expression changes, we used the expression level in rhesus macaque as an estimate of the ancestral gene expression level for human and chimpanzee. Based on this estimate, we inferred the linage-specific change for human and chimpanzee by calculating the species-specific expression difference for human as d_H_ = *μ_h_* −*μ_r_* and for chimpanzee as d_C_ = *μ_c_* −*μ_r_*. Figure 1 of the paper shows the distribution of these values. As our estimate of the ancestral expression level relies on the unrealistic assumption that there has been no change of expression level in rhesus macaque or in the common ancestor of human and chimpanzee, we repeated this analysis by retaining only genes for which the rhesus macaque expression level is an intermediate between the human and chimpanzee expression levels (where this assumption is perhaps more reasonable). Examples of these values, for the liver data, are plotted in Figures S9 and S10, where we infer that expression level has increased in the human and chimpanzee lineages in 2004 and 2141 genes, respectively.

**Consistency between studies**

Consistency between studies/platforms increases our confidence in the data and analyses. The current multi-species Nimblegen array includes probes for 816 genes that we previously studied in primate livers, using our pilot cDNA array [10]. Hence, we compared inter-species differential expression estimates in the liver between the two studies. Although the two platforms use different gene-probes, the pilot and the current gene expression data were collected using liver samples from *different* human, chimpanzee, and rhesus macaque individuals, and the studies were performed in different labs - by different people, we find a high level of consistency: The correlation of log expression ratios between the platforms is 0.47 and 0.37 for human to chimpanzee and human to rhesus macaque, respectively (Figure S11). When only genes that were identified as significantly differentially expressed in either platform are considered, the correlation increases to 0.52 and 0.48 for human to chimpanzee and human to rhesus macaque ratios, respectively. As typical correlations between array platforms in controlled experiments, using the same RNA samples, is ~0.70 [11], the level of consistency across studies in this case is satisfying. We also note that, as we discuss in the paper, we confirm principle findings that were observed using the cDNA pilot data (and a much smaller number of genes), such as the enrichment of human transcription factors whose regulation evolves under directional selection.

### Identifying genes with high level of between-individual variance in gene expression

As discussed in the paper, an important aspect of our approach to identify genes whose regulation evolves under natural selection is to examine the level of between-individual variance in expression levels across genes. This between-individual variance is the variance associated with *α_i_*, the individual random effect in the full linear model. As can be seen in Figure S12, the distribution of the between-individual variance is skewed towards lower values, with relatively small proportion of genes having higher variance. In order to classify genes as having low or high between-individual variance we fitted two straight lines to the two (approximately) linear parts of the distribution, and used the point of their intersection as the cutoff. The cutoff was set at 0.4 for liver and kidney, and at 0.3 for heart.

### Identifying genes whose regulation evolves under natural selection

To identify genes whose regulation likely evolves under *stabilizing* selection in the three primate species, we used two criteria. First, we wanted to exclude genes with evidence for DE between species (as such a pattern is not consistent with stabilizing selection on gene expression levels). To so do, we used a likelihood ratio (LR) test to test the null hypothesis that there are no expression differences between species (i.e. *μ_H_*=*μ_R_*=*μ_C_)*. Under the null hypothesis, −2∙(log-likelihood ratio) of the fits of the reduced and full model has an approximate χ^2^ distribution on 2 degrees of freedom. Since our goal at this step is to exclude genes that are DE between species, we retained genes where this statistic was less than 6 (corresponding to *P* > 0.05). Among the genes that are *not* DE between species, those whose regulation evolves under stabilizing selection are expected to have low between-individual variance. Figure S13 illustrates examples of expression patterns that are consistent with such expectation. We used the cutoffs described in the previous section, to identify genes with low variance between individuals and ranked the remaining genes by their between individual variance.

Finally, we excluded genes that had very low expression levels, as these might have low variance within and between species simply because they are not expressed. To do so, we calculated the average normalized log-expression level for each gene across all probes, plotted this intensity against the between-individual variance, and selected a cutoff that excluded genes within the obvious cluster of small absolute intensity values (Figure S14). Using this approach, we excluded genes with log absolute intensity values smaller than 7 for liver (23% of genes excluded), 6.7 for kidney (17% of genes excluded), and 7 for heart (26% of genes excluded).

Using this approach, we identified 3613, 3354, and 3198 genes whose regulation has likely evolved under stabilizing selection in liver, kidney, and heart, respectively (Table S1).

To find genes whose regulation likely evolved under *directional* selection in humans, we focused on genes whose expression level has changes exclusively in the human lineage, and maintain low within-species variance. Figure S15 illustrates examples of expression patterns that are consistent with such expectation. To identify these patterns we used three criteria. First, we excluded genes that are DE between the non-human primates. To do so, we constructed a reduced model to test if the chimpanzee and rhesus macaque expression levels are similar (i.e., *μ_C_* =*μ_R_*), and compared the maximum likelihood estimate to that of the full model. Genes that are differentially expressed between chimpanzee and rhesus macaque will have a high likelihood ratio; therefore we excluded them from further analyses (using a LR test cutoff of 2). The remaining genes have a consistent expression level in the non-human primates. Among these, we selected genes that have a significantly different expression level in humans, by using a second LR test. Here, we tested a model that reflects the assumption of similar expression levels in chimpanzee and rhesus macaque (*μ_C_* =*μ_R_*) against a null model that reflects the assumption of similar expression for all species (*μ_H_*=*μ_R_*=*μ_C_*), this time retaining genes for which we could reject the null (using an LR test cutoff of 10). Using this approach we identified 928, 856, and 1053 genes whose regulation has likely evolved under directional selection in the human liver, kidney, and heart, respectively (Table S1). When we repeated the same procedure to identify genes whose regulation has likely evolved under directional selection in chimpanzee, we identified 686, 774, and 761 such genes in liver, kidney, and heart, respectively.

**Gene classifications**

To look for enrichments in particular functional or phenotypical categories, we classified all the genes on the array into specific functional categories of interest. In order to accurately associate genes in our list with functional annotations published elsewhere, we used all known gene symbols and aliases from the kgAlias Table at the UCSC Genome Database.

We first downloaded annotations and ontology files from the Gene Ontology (GO) database (geneontology.org), and identified the terms associated with genes included on the array. We found GO information for 12100 (70.2%) of the 17231 genes included in our analysis. Genes were categorized as transcription factors (TFs) if they were associated with the GO term “transcription regulator activity” (GO:0030528 in the molecular function ontology), or with any of the successor terms in the GO graph. Similarly, genes were categorized as metabolic genes using the term GO:0008152 (“metabolic process”). In addition, we used a list of manually curated annotations of human transcription factors, prepared by Kevin White’s group, and available upon request (in the different Tables, these genes are referred to as “validated transcription factors”).

To classify genes that are associated with human disease we used annotations based on the hand-curated version of the OMIM database compiled by [12], and included annotations for somatic or germline mutations have been causally implicated in cancer (the complete working list available from <http://www.sanger.ac.uk/genetics/CGP/Census/> on Feb. 13 2007). We also included annotations for sets of genes associated with a variety of metabolic disorders (hypertension, type 2 diabetes, obesity, dyslipidemia, and metabolic syndrome), taken from Tables S1-S5 in [13].

Finally, we downloaded information on the pathways from KEGG ([www.genome.jp/kegg/](http://www.genome.jp/kegg/)).

**Analysis for enrichments of functional categories and pathways**

In order to identify functional categories and pathways that are enriched among genes with either high or low between individual variance in gene expression, we applied either a Fisher Exact Test (FET)– using 2×2 contingency Tables, or a Mann-Whitney test, using ranks (e.g., the rank of the between individual variance). We excluded from this analysis, and the following ‘enrichment’ analyses genes that do not have a record in GO, in order to avoid biasing the results with enriched functional categories that simply have more genes with studied/known functions.

In order to identify functional categories and pathways that are enriched among genes whose regulation has likely evolved under natural selection, we defined (for each tissue) the following three mutually exclusive gene groups: (i) genes whose regulation has likely evolved under directional selection, (ii) genes whose regulation has likely evolved under stabilizing selection, and (iii) all other genes not in (i) or (ii). Genes with high between-individual variance were excluded from group (iii) because they can never be included in groups (i) or (ii) (including these genes in group (iii) may bias the results). To test for enrichment we performed a two-tailed FET (using the fisher.test function).

For the GO analysis, we initially only asked for enrichment of transcription factors and/or metabolic genes (where we have a strong prior given previous studies, including our own). We did not ask about any other GO functional category and therefore did not correct our *P*-values for multiple tests.

As a second step, we performed a global analysis of enrichment in all GO categories under ‘biological processes’ and ‘molecular function’ using DAVID (<http://david.abcc.ncifcrf.gov/>). The results of this analysis are provided in Table S7. We note that a global analysis of all GO terms is somewhat difficult to interpret, because many of the functional annotations in GO are not mutually exclusive at any level of the hierarchy, and are often not very informative. That said, it can be seen in Table S7 that many of the top results are GO terms related to gene regulation and metabolic pathways, and in particular when we put together all genes whose regulation is inferred to have likely evolved under directional selection, the two top enriched GO terms are ‘transcription factor binding’ (GO:0008134) and ‘metabolic processes’ (GO:0008152). Thus, the results of the global GO analysis are consistent with our observation that transcription factors and genes in metabolic pathways are enriched among genes whose expression patterns have changed exclusively in the human lineage.

**Analysis of d_N_/d_S_**

In order to estimate d_N_/d_S_ we used high quality human-rhesus macaque alignments taken from 10,376 1:1:1 orthologous alignments between human, chimpanzee, and rhesus macaque [3]. We estimated d_N_/d_S_ for each gene using the PAML package [14], with the default parameters for nuclear DNA. We excluded cases where synonymous divergence was 0, and set d_N_/d_S_ to 0 when non-synonymous divergence was 0.

We then tested whether d_N_/d_S_ distributions are different between groups of genes (e.g. genes whose regulation has likely evolved under stabilizing selection and genes whose regulation has likely evolved under direction selection) using a Kolmogorov-Smirnov test (using the ks.test function). To test whether the medians of d_N_/d_S_ differed between groups we used a permutation test on the difference between the two medians (*D*). Specifically, we randomly divided all the values into two groups of same sizes as observed, and calculated the medians of the random groups. This permutation was repeated 10,000 times, and each time the difference between the medians of the two randomly selected groups (*D*_i_) was recorded. The test p-value was defined as the number of times where *D*_i_ ≥ *D*, divided by 10,000.

### Enrichment in chromosomal rearrangements

In order to look at enrichment of DE genes between human and chimpanzee in known chromosomal rearrangements, we first looked for an enrichment of DE genes on chromosome 2. The human chromosome 2 was created by a fusion of two independent chimpanzee chromosomes (2a and 2b), and represent the largest chromosomal rearrangement known in humans. We hypothesized that genes located on chromosome 2 are enriched with DE genes between human and chimpanzee, and tested this hypothesis using a FET.

Next, we tested whether such enrichment also exists in proximity to other large-scale rearrangements. We used the chromosomal positions of nine known large-scale rearrangement between human and chimpanzee available in Table A8 in [15]. We converted the positions from human genome build 34 (in which they were given) to build 36 using the liftOver tool (<http://genome.ucsc.edu/cgi-bin/hgLiftOver>). For one rearrangement on chromosome 15 we were unable to find corresponding coordinates in the latest genome build, and so it was not included in our analysis. The rearrangements chromosomal coordinates can be found in Table S6. For each inversion, we divided the genes on the chromosome into two groups: (i) all the genes located within 10MB of each of the rearrangement’s ends, and (ii) all other genes on the chromosome. We tested, using a FET, for enrichment of DE genes in group (i).

**Correction for multiple testing**

In order to search for patterns in the data we performed a large number of tests, as detailed above. To address the issue of multiple testing, all reported *P*-values for testing null hypothesis regarding expression differences between tissues or species were corrected using the FDR approach [9].

When we tested for enrichment of functional categories and pathways, as a first step, we focused only on transcription factors, cancer-related, and metabolic-related genes and pathway. Indeed, based on our previous pilot results [10], we had a strong prior that genes with these functions would be enriched among genes whose regulation evolves under natural selection. Given this prior and the initial focus on these functional categories and pathways, the reported relevant *P*-values need no correction. Conservatively, we give two sided *P*-value for these tests.

Next, however, we shifted our attention to look for other enrichment in the data, using all available information on pathways. A large number (>100) of different tests were performed for each comparison as part of this analysis. Nevertheless, it is unclear how to correct the relevant *P*-values, as pathways are not mutually exclusive (i.e., genes are annotated in more than a single pathway), and patterns of expression across genes are not independent. Thus, we decided to report the five most significant *P*-values in each case (as approaches for correcting for multiple tests do not affect the order of the *P*-value). We note that as the number of genes in each pathway is small, these tests have low power to detect enrichments, resulting in *P*-values that mostly would not remain significant after standard corrections for multiple testing. Thus, *P*-values reported for enrichments of functional categories and pathways other than those we had strong priors for (see above), should be taken with caution. The best use of these findings is to inform priors and raise specific hypothesis that should be tested in an independent study.

### References

1. Kent WJ (2002) BLAT--the BLAST-like alignment tool. Genome Res 12: 656-664.

2. and Analysis ConsortiumThe Chimpanzee S (2005) Initial sequence of the chimpanzee genome and comparison with the human genome. Nature 437: 69-87.

3. Gibbs RA, Rogers J, Katze MG, Bumgarner R, Weinstock GM, et al. (2007) Evolutionary and biomedical insights from the rhesus macaque genome. Science 316: 222-234.

4. Tatusova TA, Madden TL (1999) BLAST 2 Sequences, a new tool for comparing protein and nucleotide sequences. FEMS Microbiol Lett 174: 247-250.

5. Balashova VA, Abdulkadyrov KM (1984) Cellular composition of hemopoietic tissue of the liver and spleen in the human fetus. Arkh Anat Gistol Embriol 86: 80-83.

6. Brodal A (1983) The perihypoglossal nuclei in the macaque monkey and the chimpanzee. J Comp Neurol 218: 257-269.

7. Ritchie ME, Silver J, Oshlack A, Holmes M, Diyagama D, et al. (2007) A comparison of background correction methods for two-colour microarrays. Bioinformatics 23: 2700-2707.

8. Oshlack A, Chabot AE, Smyth GK, Gilad Y (2007) Using DNA microarrays to study gene expression in closely related species. Bioinformatics.

9. Benjamini Y, Hochberg Y (1995) Controlling the False Discovery Rate: a Practical and Powerful Approach to Multiple Testing. Journal of the Royal Statistical Society B 57: 289-300.

10. Gilad Y, Oshlack A, Smyth GK, Speed TP, White KP (2006) Expression profiling in primates reveals a rapid evolution of human transcription factors. Nature 440: 242-245.

11. Shi L, Reid LH, Jones WD, Shippy R, Warrington JA, et al. (2006) The MicroArray Quality Control (MAQC) project shows inter- and intraplatform reproducibility of gene expression measurements. Nat Biotechnol 24: 1151-1161.

12. Blekhman R, Man O, Herrmann L, Indap A, Kosiol C, et al. (2008) Natural selection on genes that underlie human disease susceptibility. Current Biology in press.

13. Hancock AM, Witonsky DB, Gordon AS, Eshel G, Pritchard JK, et al. (2008) Adaptations to Climate in Candidate Genes for Common Metabolic Disorders. PLoS Genetics 4: e32.

14. Yang Z (1997) PAML: a program package for phylogenetic analysis by maximum likelihood. Comput Appl Biosci 13: 555-556.

15. Marques-Bonet T, Sanchez-Ruiz J, Armengol L, Khaja R, Bertranpetit J, et al. (2007) On the association between chromosomal rearrangements and genic evolution in humans and chimpanzees. Genome Biol 8: R230.
